# Analysis of Allelic Imbalance in Rice Hybrids Under Water Stress and Association of Asymmetrically Expressed Genes with Drought-Response QTLs

**DOI:** 10.1186/s12284-016-0123-4

**Published:** 2016-09-26

**Authors:** Nelzo C. Ereful, Li-Yu Liu, Eric Tsai, Shu-Min Kao, Shalabh Dixit, Ramil Mauleon, Katrina Malabanan, Michael Thomson, Antonio Laurena, David Lee, Ian Mackay, Andy Greenland, Wayne Powell, Hei Leung

**Affiliations:** 1 Genetics and Biotechnology Division, International Rice Research Institute (IRRI), Los Baños, Laguna Philippines; 2 Department of Agronomy, National Taiwan University (NTU), Taipei City, 100 Taiwan; 3 Institute of Plant Breeding, University of the Philippines, Los Baños, Laguna Philippines; 4 The John Bingham Laboratory, National Institute of Agricultural Botany (NIAB), Huntingdon Road, Cambridge, CB3 0LE UK; 5 Crop Science Cluster, College of Agriculture, University of the Philippines, Los Baños, Laguna 4031 Philippines; 6 Texas A &M, Department of Soil and Crop Sciences 2474 TAMU, College Station, TX 77843-2474 USA; 7 SRUC, Peter Wilson Building, West Mains Road, Edinburgh, EH9 3JG UK

**Keywords:** Rice (*Oryza sativa* L.), Allele-specific expression (ASE), Allelic imbalance (AI), Drought, RNA-seq, Selective genotyping, Co-localization analysis, Quantitative trait loci (QTL)

## Abstract

**Background:**

Information on the effect of stress on the allele-specific expression (ASE) profile of rice hybrids is limited. More so, the association of allelically imbalanced genes to important traits is yet to be understood. Here we assessed allelic imbalance (AI) in the heterozygote state of rice under non- and water-stress treatments and determined association of asymmetrically expressed genes with grain yield (GY) under drought stress by *in-silico* co-localization analysis and selective genotyping. The genotypes IR64, Apo and their F1 hybrid (IR64 × Apo) were grown under normal and water-limiting conditions. We sequenced the total RNA transcripts for all genotypes then reconstructed the two chromosomes in the heterozygote.

**Results:**

We are able to estimate the transcript abundance of and the differential expression (DE) between the two parent-specific alleles in the rice hybrids. The magnitude and direction of AI are classified into two categories: (1) symmetrical or biallelic and (2) asymmetrical. The latter can be further classified as either IR64- or Apo-favoring gene. Analysis showed that in the hybrids grown under non-stress conditions, 179 and 183 favor Apo- and IR64-specific alleles, respectively. Hence, the number of IR64- and Apo-favoring genes is relatively equal. Under water-stress conditions, 179 and 255 favor Apo- and IR64-specific alleles, respectively, indicating that the number of allelically imbalanced genes is skewed towards IR64. This is nearly 40–60 % preference for Apo and IR64 alleles, respectively, to the hybrid transcriptome. We also observed genes which exhibit allele preference switching when exposed to water-stress conditions. Results of *in-silico* co-localization procedure and selective genotyping of Apo/IR64 F_3:5_ progenies revealed significant association of several asymmetrically expressed genes with GY under drought stress conditions.

**Conclusion:**

Our data suggest that water stress skews AI on a genome-wide scale towards the IR64 allele, the cross-specific maternal allele. Several asymmetrically expressed genes are strongly associated with GY under drought stress which may shed hints that genes associated with important traits are allelically imbalanced. Our approach of integrating hybrid expression analysis and QTL mapping analysis may be an efficient strategy for shortlisting candidate genes for gene discovery.

**Electronic supplementary material:**

The online version of this article (doi:10.1186/s12284-016-0123-4) contains supplementary material, which is available to authorized users.

## Background

Studies reveal that heritable variations do not reside in protein-coding DNA alone but also in the regulatory sequences. It is now increasingly clear that DNA segments coding for proteins account for only one aspect of heritable variations.

Non-coding variants, gene imprinting, epigenetic factors, *trans*-acting elements, among others were revealed to play important roles in variations and diversity. Even an alteration in one or more nucleotides in the *cis-*acting regulatory elements residing in non-coding regions of the DNA contributes to phenotypic variations. It can alter the binding of a transcription factor (TF) affecting transcription initiation, rate and stability which consequently brings about asymmetric expression of alleles residing in one genome, a phenomenon called allele-specific expression (ASE) imbalance or simply allelic imbalance (AI) (Syvänen et al. 2007; Heap et al. 2010; Bell and Beck 2009).

Preferential expression of one allele over another can be detected by differences in transcript number. ASE is ascribed to *cis-*acting polymorphism, *cis-*acting epigenetic effectors, or a combination of both genetic and epigenetic effectors as *trans*-acting factors have an equal opportunity to affect both alleles within a nucleus (Bell and Beck 2009; Guo et al. 2015).

AI has been the subject of several studies in humans (some are described by Syvänen et al. 2007; Tsuchiya et al. 2009; Voutsinas et al. 2010) and mouse hybrids (Crowley et al. 2015). A review of the research in AI and its regulatory mechanism was published by Gaur et al. (2013). It has been likewise reported to occur in several crop hybrids such as maize (Springer and Stupar, 2007a), barley (von Korff et al., 2009), and rice (Guo et al. 2015). A recent report showed that ASE exists in the natural populations of *Cirsium arvense*, an invasive Compositae weed (Bell et al. 2013). In maize, a review showed the potential contributions of allelic variation to heterosis (Springer and Stupar, 2007b) and the prevalence of *cis*-acting regulatory variations which contribute to biased allelic expression (Springer and Stupar, 2007a).

Only a limited number of studies on AI in rice has been conducted and hence it is still poorly understood. He et al. (2010) found that gene activity was correlated with DNA methylation and both active and repressive histone modifications in the transcribed region. Using SNPs as markers, they observed a high correlation of allelic bias of epigenetic modifications or gene expression in reciprocal hybrids with differences in the parental lines. The study concluded that transcriptional and epigenetic trends in reciprocal rice hybrids contribute to heterosis.

A similar study by Wang et al. (2011) indicated that drought-induced genome-wide DNA methylation changes accounted for ~12.1 % of the total site-specific methylation differences in the rice genome. These epigenetic changes can be considered as an important regulatory mechanism for rice plants to adapt to drought and other environmental stresses.

A recent study on epigenomic analysis using ChIP-seq on *indica-indica* hybrids concluded that histone modifications regulate AI (Guo et al. 2015). This study confirms the contribution of epigenetic mechanism in AI. Earlier studies in rice also suggested that *trans* effects mediate the majority of the transcriptional differences in hybrid offspring and to a lesser extent, intergenerational epimutations (Chodavarapu et al. 2012). These studies in rice showed the important role of *trans*-acting factors on asymmetric expression of parent-specific alleles. However, little is known about ASE in rice under contrasting environmental conditions.

Here we identified genes exhibiting AI under two different water regimes and demonstrated their association with drought-yield QTLs. We reasoned that by exposing the hybrids to two contrasting conditions, we can identify genes that exhibit AI and condition-mediated changes in their ASE profiles. We hypothesized that such genes can be highly associated with drought-tolerance response and can be linked with previously identified drought-response QTLs in the same genetic background.

Specifically, we measured AI in the F1 hybrids and determined changes in ASE profiles of genes between non- and water-stress treatments in the *indica-indica* genetic background using the two varieties IR64 and Apo (IR55423-01). IR64 is a high-yielding variety but was previously shown to be moderately susceptible to drought conditions. Apo, on the other hand, is a drought-tolerant indica variety.

Our study is limited to one-way hybrid cross (IR64 × Apo) exposed to two contrasting water regimes to provide cross-specific information on the effect of stress on allelic imbalance then assess the association of asymmetrically expressed genes with previously identified QTLs known to be involved in drought response. We employed RNA-seq platform to: (1) identify genes asymmetrically expressed between the two genotype-specific alleles in the hybrid in non- and water-stress conditions, (2) assess the level of expression and parental allele preference (magnitude and direction, respectively) of AI on a genome-wide scale, (3) compare the ASE profile of genes under non- and water-stress conditions and then assess changes in expression patterns as effected by the two treatments, and (4) assess the participation of AI genes on drought response.

## Results and Discussion

Using RNA-seq, AI was assessed in one-way hybrid cross in rice after exposure to non- and water-stress conditions. Water-stress treatment was initiated at the early stage of flowering after which collection of leaf samples was performed. This is the stage when rice is most sensitive to drought consequently affecting grain yield (Boonjung and Fukai, 1996). Genes identified to exhibit asymmetric expression using AI assay were further tested for their association with drought-yield QTLs by *in-silico* colocalization procedure and selective genotyping.

To generate the hybrids, two genotypes with contrasting response to water-limiting conditions were crossed: (i) IR64, a popular high-yielding drought-susceptible *indica*, and (ii) Apo (IR55423-01), an upland drought-tolerant *indica*. Both materials have been used in previous studies on selection for yield (Venuprasad et al. 2007; Venuprasad et al. 2008) and QTL mapping (Venuprasad et al. 2009) under drought stress. We sequenced the RNA of twelve samples which includes the two parental genotypes and the hybrids exposed to two contrasting treatments (3 genotypes × 2 treatments × 2 replicates) using Illumina platform (described in the Materials and Methods) and generated a pseudo-molecule based on the *O. sativa* MSU7 cDNA (Nipponbare, a *japonica*). A pseudo-reference sequence was created for the subsequent data analysis because of the sequence variations between *japonica* and *indica* (i.e. reference genome versus our parental *indica* genotypes, respectively).

### Construction of a Pseudo-Reference Sequence

To create a pseudo-reference sequence, SNPs and InDels were identified between *japonica* and *indica*. After mapping the reads from the parental genotypes Apo and IR64 (4 samples each) to the MSU7 cDNA sequences, we found 106,884 sites (SNPs) which are common to both Apo and IR64 but different from those from the reference, and 8340 sites (InDels) which are common insertions or deletions in Apo and IR64 but different from the reference. These variations imply that there are about 0.92 SNPs and 0.07 InDels per kilobase. The identified SNPs and InDels in this step were used to replace the corresponding nucleotides in the reference MSU7 cDNA sequences to construct the pseudo-reference sequence. The alignment rates of the reads generated from the 12 samples which mapped against the pseudo-reference range from 67.21 to 85.31 % while the alignment rates range from 66.11 to 84.19 % if the reads were mapped against the original MSU7 cDNA sequences. These results show a 0.7–1.6 % improvement rates (Table 1).

**Table 1:** Comparison of the mapping rates using the reads generated from the samples as query sequence and the original MSU7 cDNA reference and the pseudo-reference as the subject sequences

Treatments		Rep 1			Rep 2		
		Original MSU7 (%)	Pseudo-reference (%)	Improvement (%)	Original MSU7 (%)	Pseudo-reference (%)	Improvement (%)
Control	Apo	77.42	78.43	1.01	84.19	85.31	1.12
	IR64	77.92	79.03	1.11	78.07	79.68	1.61
	F1	76.65	77.77	1.12	83.78	85.23	1.45
Stress	Apo	78.8	79.91	1.11	83.53	84.7	1.17
	IR64	66.11	67.21	1.1	70.28	71.75	1.47
	F1	78.34	79.11	0.77	81.01	82.6	1.59

We also mapped the reads of the *indica* rice variety 93-11 from Beijing Genomics Institute (BGI) against the original MSU7 cDNA sequences and the pseudo-reference. Their alignments yielded 70.06 % and 71.15 % mapping rates, respectively. These figures indicate an improvement of the mapping rate by 1.09 % which is approximately the same as that of the observed values from our 12 samples. The increase in the alignment rates shows that extra information was incorporated in the pseudo-molecule sequences by modifying the SNP and InDel sites.

### Identification of SNPs to Identify the IR64 and Apo Alleles

In this study, the SNPs were used as molecular markers to identify which alleles in the hybrids belong to which parental genotypes. The reads from the eight samples of Apo and IR64 were mapped against the pseudo-reference by bowtie2 (Langmead and Salzberg, 2012). We found 48,175 sites which showed polymorphism between Apo and IR64. This modest level of polymorphism may be possibly due to the similarity of the two genomes since both are *indica*. Low level polymorphism had been previously reported between parents belonging to the same ecotypes (Ali et al. 2000).

Using these 48,175 SNPs, we were able to identify which SNP alleles in the hybrids belong to which parental genotypes. We refer our strategy of finding parent-specific alleles in the hybrids as ‘read-wise’ which is described in the Materials and Methods. Under normal conditions, 2.56 % and 5.41 % of the F1 reads from reps 1 and 2, respectively, can be identified as either IR64- or Apo-specific alleles. Under water-stress conditions, 1.52 % and 5.23 % of the F1 reads from reps 1 and 2, respectively, can be identified (Table 2). The F1 reads with known parental origins were further analyzed for AI.

**Table 2:** Number of reads which can be assigned as IR64- or Apo-specific allele based on parental SNPs

	Rep 1			Rep 2		
	F1 Mapped Read Counts			F1 Mapped Read Counts		
Control	21,860,178	From Apo	276,827	51,364,894	From Apo	1,378,264
		From IR64	282,901		From IR64	1,403,493
		Unspecified	21,300,450		Unspecified	48,583,137
Stress	36,853,236	From Apo	275,132	54,525,736	From Apo	1,399,485
		From IR64	288,589		From IR64	1,454,190
		Unspecified	36,289,515		Unspecified	51,672,061

A modest percentage level of F1 reads containing information on their parental origins was obtained due to the low densities of parental SNPs (48175/66338 = 0.72 SNPs per gene on average), i.e. between Apo and IR64 transcriptomes. Most of the sequence reads do not show SNPs between the parents and therefore were discarded for further analysis on AI.

### Genome-Wide Analysis of ASE in the F1

The magnitude and direction of ASE of genes in the rice hybrids were estimated and classified into two categories depending on the fold change (FC) values between Apo and IR64 (Apo/IR64) the cross-specific paternal and maternal alleles, respectively. These categories include: (1) symmetrically or biallelically expressed, and (2) asymmetrically expressed or imbalanced genes. Symmetrical expression happens when genes have relatively equal expression between the two genotype-specific alleles in the hybrids at fold expression ratio lower than 1.25×. Because both alleles are expressed at a relatively equal magnitude, the expression is biallelic. The minimum fold value of 1.25× has been used previously (Bell et al. 2013; McManus et al. 2010) and will be widely used in this paper. Asymmetrical or imbalanced, on the other hand, happens when the allelic expression fold difference between the two alleles is equal to or more than 1.25×. This can be further classified into two sub-categories: IR64- and Apo-favoring genes. The former type happens when IR64-specific allele of the same locus supersedes the expression of the other by at least 1.25 fold; the latter when the reverse happens.

This classification is similar to a previous report (Song et al. 2013). However, the maternal and paternal allelic contributions were effectively estimated in the present study while the former report could not distinguish the parental alleles in the hybrids mainly due to technological limitations at that time. Furthermore, monoallelic expression was not significantly observed in our study. This happens when only one of the two parent-specific alleles is expressed.

In this study, some genes have been identified to have more than one splice variant as a consequence of alternative splicing. Thus, genes will be represented in terms of gene models or transcript variants as described in MSU Rice Genome Annotation located at http://rice.plantbiology.msu.edu/.

#### AI at 1.25× Fold Expression

Using our pipeline, we found 2337 and 2092 genes with SNPs between the two genotype-specific alleles in the hybrid under non- and water-stress conditions, respectively (Additional file 1: Table S1 and Additional file 2: Table S2). The modest number of genes is due to the limited variations between the two parental genomes in the heterozygote. Of these, only 560 genes exhibited significant DE between the two alleles under normal conditions at *P* < 0.05 (Additional file 3: Table S3). This corresponds to 24.0 % of the total number of genes detected (560/2337). On the other hand, under water-stress conditions, 676 genes showed significant DE between the two genomes in the heterozygous state which corresponds to 32.2 % of the total number of genes (676/2092) (Additional file 4: Table S4). These results indicate that a higher number of genes significantly differentially expressed between the two alleles are induced in response to water-stress conditions.

Under non-stress treatment, 179 and 183 genes favorably expressed Apo- and IR64-specific alleles, respectively (Table 3) using a 1.25× fold expression ratio as threshold. These values show that under normal conditions, the number of genes favoring each parental allele in the heterozygote was about equal. This suggests that AI is inherent to the organism, not necessarily induced by stress. There are 198 genes biallelically expressed under normal conditions. This means that both parental alleles at the same locus are expressed in a relatively equal copy number. Additional file 5: Figure S1A was generated to show genes in the F1 hybrids exhibiting ASE under non-stress conditions.

**Table 3:** Number of genes exhibiting preferential expression for Apo- and IR64-specific alleles in the hybrids exposed to two contrasting conditions at a minimum fold differences of 1.25× and 2.0× (at *P* < 0.05)

Description	For at least 1.25-fold		For at least 2.0-fold	
	Non-stress	Water-stress	Non-stress	Water-stress
No. of genes which prefer Apo-specific allele	179	179	77	69
No. of genes which prefer IR64-specific allele	183	255	80	95
Total no. of genes exhibiting AI	362	434	157	164
% genes exhibiting AI (over total number of genes significantly DE at *P* < 0.05)	64.6	64.2	28.0	24.3

On the other hand, 179 and 255 genes favored the expression of Apo- and IR64-specific alleles, respectively, under water deficit (Table 3; Additional file 6: Figure S1B). These figures correspond to 41 %-59 % (nearly 40-60) preferential expression for Apo and IR64 alleles (at 1.25× fold-expression threshold and *P* < 0.05), respectively, for nuclear genome. These figures do not account for the contribution of organellar genomes (i.e. mitochondria and plastids). There appears to be a bias expression of IR64- (maternal) over Apo-specific allele (paternal) for this specific hybrid cross. These data showed that water-stress conditions skewed preferential expression towards IR64-specific allele, the cross-specific maternal allele. There are 242 genes biallelically expressed under water-stress conditions. Notably, the number of genes (179) favorably expressing Apo-specific allele under non- and water-stress conditions remained unchanged; only the number of genes preferentially expressing IR64-specific allele increased from non- to water-stress conditions. Genes in the F1 hybrids exhibiting ASE under water-stress conditions are shown in Additional file 6: Figure S1B.

In total, 362 and 434 genes showed AI under normal and stress conditions, respectively, demonstrating a higher number of genes asymmetrically expressed under water-stress conditions. However, if we compute the number of genes exhibiting AI over the total number of transcripts significantly differentially expressed, 64.6 % are imbalanced under normal condition (Table 3). This ratio does not significantly deviate from the ratio obtained under stress conditions (64.2 %). This suggests that the stress did not change the ratio of genes exhibiting asymmetric expression over the total number of genes identified to have significant DE between the two alleles. The stress only enhances gene expression and skews the number of AI genes toward the IR64 allele, the cross-specific maternal allele.

A summary of results on AI is shown in Table 3. Heat maps were also generated to show the allele-specific preference of each F1 gene under non- and water-stress conditions (Additional file 5: Figure S1A and Additional file 6: Figure S1B).

#### DE at 2.0× Fold Expression

If we are to impose a minimum FC of 2.0×, 77 and 80 showed preferential expression for Apo- and IR64-specific alleles, respectively, under non-stress conditions. A 2.0× fold minimum expression difference was used in previous studies (Song et al. 2013; Paschold et al. 2012). These data show that 157 genes (28.0 %) are asymmetrically expressed under normal conditions. There are 403 and 512 biallelically expressed genes under normal and water-stress conditions, respectively. Under water-stress conditions, of the 676 genes, 69 preferentially expressed for the Apo-specific allele, two of which are monoallelically expressed, and 95 preferentially expressed the IR64-specific allele. This further affirms that water-stress skews the number of genes in favor of the IR64-specific allele, a bias expression towards the cross-specific maternal allele.

If we consider 2.0× fold expression ratios, these ratios (28.0 % and 24.3 %, under non- and water-stress, respectively) are lower than the values reported in the literature. However, if the cut-off is set at 1.25× fold, these ratios are more consistent with those reported previously. A study in barley showed that 63 % of genes (19/30) tested showed allelic expression imbalances (von Korff et al. 2009). In maize, 43-53 % of the 316 analyzed genes (depending on the cross and tissues) showed unequal allelic expression (Springer and Stupar, 2007b). A similar study on genome-wide ASE analysis using massively parallel signature sequencing showed that 60 % of the genes in the maize hybrid meristems exhibited differential allelic expression (Guo et al. 2015). Additionally, different approaches to measure ASE may influence these variations (von Korff et al. 2009).

**Fig. 1:**
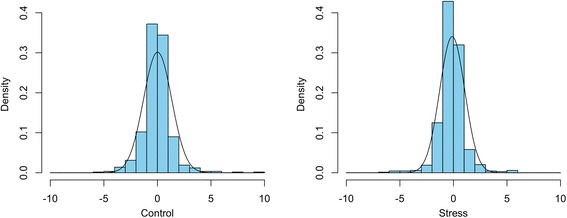
Genome-wide analysis of ASE in F1 at (left) non- and (right) water-stress conditions (for 2× fold minimum expression). The right side of both graphs starting from the origin 0 (positive) shows FC values when Apo-specific allele is preferentially expressed over IR64-specific allele; the left side (negative values) when IR64-specific allele is preferentially expressed over Apo-specific allele

In summary, the hybrid expresses higher number of genes significantly differentially expressed between the two alleles (at *P* < 0.05) during water-stress conditions as compared to during normal conditions indicating that water stress enhances gene expression. Our results further suggest that genes exhibiting AI is inherent to the organism regardless of the conditions and is not a consequence of stress. On a genome-wide scale, the number of genes favoring each of the parental alleles approaches a normal distribution curve (using 2.0× fold minimum level of expression; *P* < 0.05) (Fig. 1). However, at water-limiting conditions, the distribution curve is skewed towards IR64-specific allele – a global distortion of preferential expression. This indicates that more genes favor the maternal over the paternal allele under stress conditions in this particular hybrid cross. It is surprising to note that while Apo is the drought-tolerant genotype, IR64-specific allele is preferentially expressed in the hybrid. This is contrary to our initial speculation that the tolerant genotype (Apo) should exhibit preferential expression in the heterozygote.

We speculate that this genome-wide change is a mechanism to respond to water-limiting conditions and that *trans*-sensory effectors may have contributed in the global distortion favoring IR64 allele. This was earlier reported by Tirosh et al. (2009) in which *trans* effects were condition-dependent and that expression difference in *trans* reflected a differential response to the environment. F1 hybrids exposed under two contrasting treatments have theoretically the same structural sequences including *cis*-acting elements, thus these regulatory regions may not contribute to the expression differences between the samples. While *trans*-sensory effectors may have contributed on these changes, epigenetic factors cannot be entirely excluded as it was earlier reported (Wang et al. 2011).

Whether the genome-wide preferential expression as effected by water-limiting conditions is maternal parent-of-origin or genotype-dependent is yet to be concluded. Further study using reciprocal hybrid cross is recommended to address this question.

Variations in the *cis*-acting regulatory sequences cause asymmetric expression of the two genotype-specific alleles in the hybrids because both alleles are exposed to the same pool of *trans* factors within a nuclear environment. However, changes in environmental cues may have affected *trans*-sensory effectors subsequently leading to changes in the genome-wide expression profile. The stress which acted as a “pressure” distorted the normal ASE profile toward IR64 allele. This supports previous studies that such regulatory, as well as epigenetic factors, may have mediated the interactions between the environment and the genome (Tirosh et al. 2009; Herceg 2016). Furthermore, it was earlier reported that changes in regulatory regions, *cis* and *trans*, may contribute to evolutionary changes (Wittkopp et al. 2004). However, further studies are needed to elucidate the effect of environmental pressures to evolutionary changes vis-à-vis regulatory factors and the epigenetic landscape.

#### Relative Transcript Abundance

The relative transcript abundance of the two parent-specific alleles in the F1 can now be estimated and the relative expression ratios can be derived owing to the advent of sequencing platforms and computational methods. Our data show varying expression ratios between the two transcripts of the same locus in the F1. In some genes, two alleles of the same locus are expressed symmetrically (biallelic) in which both parent-specific transcripts contributed nearly equally to the hybrid transcriptome. For several genes (listed in Additional file 3: Table S3 and Additional file 4: Table S4), one of the two alleles shows higher level of expression (dominant) over the other one with lower expression level. For example, in the gene LOC_Os01g27360.1 (glutathione S-transferase), IR64- and Apo-specific alleles contributed 201.08 and 4.75 transcript copies (after normalization and read count adjustment), respectively, to the hybrid transcriptome under non-stress conditions. This is consistent with a recent finding (Gonzàlez-Porta et al. 2013), in which most protein-coding genes in humans have one major transcript expressed at significantly higher level than others. This spectrum of ratios between the parent-specific transcripts indicates the varying possible interactions that exist between the two genomes in the hybrid.

Furthermore, the ability of the hybrids to “skew” towards a preferred allele during harsh conditions suggests that the presence of two genomes in the hybrids allow flexibility in response to stressful environments. The expression of two diverse genomes, which is demonstrated by biallelic genes, may subsequently result in a more diverse peptide population when translated. Previous studies in humans suggested that not all the transcripts that contribute to transcriptome diversity are equally likely to contribute to protein diversity (Gonzàlez-Porta et al. 2013). Jovanovic et al. (2015) cited studies indicating a considerable discrepancy between RNA and protein levels. Little is known about genotype-specific alleles in hybrids of crop species.

#### Genes with Extreme Cases of AI

An allelic expression level difference of 582.6-fold was the highest observed FC which is exhibited by LOC_Os10g24004.1 (unknown), an Apo-favoring gene (Additional file 3: Table S3). Consistently, this gene was observed to exhibit the highest expression fold difference of 63.1-fold during water-stress condition, also favoring the Apo-specific allele.

A gene encoding for SCP-like extracellular protein (LOC_Os01g28450.1) exhibited the second highest expression fold difference of 173.2× during non-stress condition. The same gene also exhibited a relatively high expression difference (47.3× fold) under water-stress condition favoring the Apo allele. On the other hand, genes encoding for glutathione S-transferase (LOC_Os01g27360.1), regulator of ribonuclease (LOC_Os02g52430.1), metallothionein-like protein (LOC_Os05g11320.1) and thaumatin (LOC_Os12g43450.1) showed the highest levels of FC (42.3-, 27.5-, 23.5- and 14.9-fold, respectively) which favor the IR64-specific allele under non-stress conditions. The gene thaumatin was previously shown to confer tolerance against fungal infection and abiotic stresses (Rajam et al. 2007).

Under water-stress condition, four splice variants of the gene encoding for jacalin-like lectin proteins (LOC_Os01g24710) were expressed favoring the Apo-specific allele. Two of these variants exhibited monoallelic expression, an extreme case of AI in which the IR64-specific allele is completely silenced. Studies in wheat suggested that these proteins are component of the salicylic acid- and jasmonic acid-dependent defense signaling pathways (Xiang et al. 2011). The other two variants of jacalin-like proteins showed the second and third highest expression difference of 50.2- and 51.8-fold favoring the Apo-specific allele under water-stress condition.

As in the non-stress conditions, the gene encoding for regulator of ribonuclease showed the highest level of FC (67.2-fold) which favor the IR64-specific allele under water-stress condition (Additional file 4: Table S4). Additionally, another thaumatin gene was found to have high expression fold difference of 22.5 × .

Notably, genes with extreme fold differences between the two alleles in the heterozygote either confer tolerance or are important components of defense systems under stress conditions. So far, we have established that the number of genes favoring Apo-specific allele is relatively equal to the number of genes preferring IR64-specific under non-stress conditions. However, in terms of the magnitude of expression, several genes strongly express the Apo-specific allele, the paternal allele for this specific hybrid cross, as shown by the high fold-differences between the two parental alleles. This difference in quantitative expression may account for the phenotypic expression of tolerance to water stress in Apo.

### ASE Profiles of Genes Between the two Conditions

We performed pairwise comparison of the ASE profiles of each gene with differentially expressed alleles between the two contrasting conditions to characterize any condition-mediated expression changes. Our method of pairwise comparison is described in the Materials and Method. We found 976 genes, of which 885 are commonly expressed in both conditions if we use a lenient approach (i.e. *P* < 0.05 in one or both conditions); 57 and 34 genes uniquely expressed under non- and water-stress conditions, respectively. Additional file 7: Table S5 shows the result of this pairwise comparison. Heatmaps were created (Additional file 8: Figure S2A, Additional file 9: Figure S2B, Additional file 10: Figure S2C, Additional file 11: Figure S2D and Additional file 12: Figure S2E) to visualize these patterns.

By comparing the ASE profiles of genes expressed at 1.25× fold difference between the two conditions (at *P* < 0.05 in one or both conditions), we are able to identify: (1) transcript Presence/Absence Variation or tPAV, (2) bi-directional and (3) unidirectional expression behaviors.

#### tPAV

There are 91 genes (9.3 % of the 976 genes) that exhibit tPAV. This happens when a gene is expressed in one condition but not in the other condition regardless of its specific allele preference. Of these, 57 are expressed only during normal conditions but are repressed during the stress conditions (tPAVnormal in Additional file 7: Table S5; Additional file 8: Figure S2A). On the other hand, 34 are induced only during stress conditions but are repressed during normal condition (tPAVstress in Additional file 7: Table S5; Additional file 9: Figure S2B).

#### Bidirectional

Only 50 genes (5.1 %) exhibited bidirectional expression. This happens when the allele preference of a gene changes from one condition to the other contrasting condition, hence a change in direction. The gene exhibits the ability to switch from one to the other allele as effected by environmental changes, hence, condition-dependent expression deviations. We speculate that *trans*-sensory and epigenetic factors may have influenced these changes.

Of these, 18 showed IR64- to Apo-specific allele change from non- to water-stress conditions, respectively. Some of these genes encode for 6-phosphofructokinase (LOC_Os01g09570), Ser/Thr protein phosphatase family protein (LOC_Os11g05400), and ultraviolet-B-repressible protein (LOC_Os03g22370.1). These changes in expression profile are illustrated in Additional file 10: Figure S2C (green cells in the normal to red cells in the water-stress conditions).

On the other hand, 32 genes showed Apo- to IR64-specific allele change, from normal to stress conditions, respectively (illustrated in Additional file 10: Figure S2C; red cells in the normal to green in stress conditions). These genes include transmembrane amino acid transporter (LOC_Os01g41420), membrane protein (LOC_Os03g51650), and RNA recognition motif (LOC_Os03g17010.1). Another splice variant of the last-named gene (LOC_Os03g17010.2) was found to exhibit tPAV(normal). Additionally, LOC_Os12g18650 has two variants with varying ASE behaviors: versions 6 and 7 exhibit unidirectional (described below) and bidirectional, respectively. These results show that splice variants of a particular gene have varying ASE behaviors with respect to changes in environmental conditions.

The identification of genes exhibiting bidirectional expression behavior may potentially demonstrate that preferential expression (and possibly dominance) for some genes is condition-mediated as a consequence of gene-environment interactions.

#### Unidirectional

If there are genes exhibiting bidirectional expression behavior, there are also genes that the organism consistently prefers in both conditions. We call this behavior “unidirectional expression.” The change in ASE profile is in the magnitude alone (expression level), not on the direction (allele preference) since the gene prefers a particular allele in both conditions. Unlike bidirectional, unidirectional genes are minimally influenced by changes in environmental cues. There are 350 (35.9 %) genes which exhibit this type of behavior. Of these, 179 consistently favored IR64-specific allele in both conditions (Additional file 11: Figure S2D); 171 favored Apo-specific allele (Additional file 12: Figure S2E).

To summarize, the changes in ASE profiles are ascribed to the presence of two different genomes in the F1 hybrid which allows dynamic expression of either or both alleles under any given condition. Two contrasting genomes in the hybrid provide a wider dynamic response to changes in environmental cues and allow greater potential adaptive advantage. The heterozygosity between the genomes lead to structural and functional diversity of peptides (depending on the functional consequences of translation) inside the cell or tissues which may allow the organism to respond to stress in a way that may not be possible otherwise. Tolerance in this manner is attributed to the ability of the organism to respond with environmental cues vis-à-vis condition-mediated changes in ASE behaviors. These mechanisms provide the organism to adapt to harsh conditions. Parent-specific variations in hybrids allow dynamic interactions and changes in the genome that may endow hybrids with greater ability to adapt to environmental changes or stresses. These information may potentially contribute to the genomic and epigenetic insights explaining heterosis.

### *In-Silico* Association of AI Genes with Drought-Response QTL

To initially test the involvement with grain yield (GY), AI genes found to respond in two contrasting water regimes (listed in Additional file 7: Tables S5) were aligned *in-silico* with genetic markers identified to have significant effects on GY under drought stress (Venuprasad et al. 2009). These molecular markers were monitored in the same parental backgrounds (Apo/IR64) as our study using F_2:3_ lines. Using Gramene (*O. sativa indica* genome), this approach of co-localization entails *in silico* anchoring the positions of the SSR markers and the AI genes to estimate their physical-genetic linkage.

This procedure identified a number of AI genes closely linked with the QTL markers known to participate in drought stress in the same parental background. Results of this co-localization analysis are summarized in Table 4. Genes that co-localize with QTL markers are distributed across six chromosomes – 1, 3, 6, 8, 9 and 12. The physical distance (in kb) between the AI genes and the co-localizing markers are estimated based on Gramene (*O. sativa indica* genome), most of which are less than 300 kb.

**Table 4:** A summary of the AI genes co-localizing with SSR markers previously identified to be associated with GY under drought stress in the study by Venuprasad et al. (2009) using the same genetic backgrounds (Apo/IR64). Markers are based on Gramene (*O. sativa indica* genome)

Chr #	SSR Name	AI genes co-localizing with the QTL marker	Description/ Function	Estimated distance between the marker and the genes (kb) in the *indica* genome
1	RM6703	LOC_Os01g59980	zinc finger	209
2	RM71	LOC_Os02g15520	transposon	67
		LOC_Os02g15660	tetratricopeptide repeat	44
3	RM3387	LOC_Os03g02020	stress responsive A/B barrel domain containing proteins	20
		LOC_Os03g53710	aldose-1-epimerase	110
	RM520	LOC_Os03g54980	expressed protein	330
		LOC_Os03g55030	UPD-gucosyl transferase	360
6	RM510	LOC_Os06g04030	Histone H3	210
8	RM256	LOC_Os08g38300	core histone H2A/H2B/H3/H4	11
		LOC_Os08g39150	expressed protein	470
		LOC_Os08g39160	formyl transferase	
9	RM201	LOC_Os09g35940	cytochrome P450	520
12	RM511	LOC_Os12g29330	No apical meristem	9

Markers coinciding with AI genes encode for important stress-response factors involved in transcription regulation and binding. Zinc finger (ZF), a known regulatory factor coincides with the marker RM6703 (139 cM) in chromosome 1. A tetratricopeptide repeat, a binding protein is closely linked with RM71 at chromosome 2 while a stress responsive alpha/beta barrel protein is adjacent to the QTL marker RM3387 at chromosome 3 (0 cM).

The DNA-binding proteins histone H3 and core histone H2A/H2B/H3/H4 are closely linked with the markers RM510 on chromosome 6 (21 cM) and RM256 at chromosome 8, respectively. The former exhibits bidirectional bias expression (Additional file 7: Table S5).

The AI gene encoding for no-apical meristem (NAM) transcription factor (LOC_Os12g29330) is closely linked to the marker RM511, with a physical distance of 9 kb. Interestingly, this is a major gene in the large-effect QTL *qDTY*_*12.1*_ region identified to be a drought-yield QTL using Vandana/Way Rarem cross (Bernier et al. 2007; Dixit et al. 2015). It is a mainstay of the intra-QTL region and plays roles on spikelet fertility and root proliferation under drought. This was found to exhibit unidirectional expression behavior which consistently prefers IR64 allele in both conditions (Additional file 7: Table S5).

In summary, Table 4 provides a number of AI genes involved in drought confirmed solely by *in silico* co-localization procedure using markers previously known to co-segregate with QTL regions. Genes identified particularly those lying closely to QTL markers are promising candidates for drought-tolerance response. The involvement of these loci in this important abiotic stress is of practical interest for breeding.

### Association of AI Genes in Drought Response by Selective Genotyping

To further validate the involvement of several AI genes with GY during drought stress, selective genotyping was performed using Apo/IR64 F_3:5_ recombinant inbred lines (RILs). These lines were phenotyped to confirm the involvement of the identified AI genes with GY under water-limiting conditions. Both tail ends which constitute 25 % of the whole population were genotyped.

QTL regions validated to participate in drought response in the co-localization procedure were further tested by adding markers contiguous to their locations. These were supplemented with additional SSR markers distributed across the genome which coincide with the AI genes. The results of the initial polymorphism survey between the parental genotypes showed a modest level of polymorphism. Of the 153 SSR markers identified to reside closely with these genes, 22 (14 %) exhibited clear polymorphism between the two parents. The parental genotypes exhibited a low level polymorphism since both lines belong to the same subspecies (*indica*).

Results of selective genotyping are shown in Table 5. Six of the 22 polymorphic markers showed significant association with GY (at varying P values of 0.05 and 0.01) under water-stress. AI genes co-localizing with the markers associated with GY were distributed across four chromosomes 1, 2, 3, and 8.

**Table 5:** AI genes which co-localize with markers confirmed to respond during drought stress by selective genotyping. These genes are potential candidates for gene discovery

Chr#	Marker	*P*	R^2^ (%)	AI genes colocalizing with the markers	Estimated distance between markers and genes (kb) in the *indica* genome
1	RM6333	**	21.12	LOC_Os01g65400 (DNA polymerase I)	52
				LOC_Os01g65650 (receptor-like protein kinase HAIKU2 precursor)	113
	RM11943	*	19.11	LOC_Os01g64960 (chlorophyll A-B binding protein)	188
				LOC_Os01g65130 (peptide transporter)	48
				LOC_Os01g65220 (XRN 5'-3' exonuclease N-terminus domain)	0.2
				LOC_Os01g65169 (proton-dependent oligopeptide transport)	25
				LOC_Os01g64790 (AP2)	306
2	RM5789	**	24.48	LOC_Os02g37030 (protein binding protein)	0.5
	RM3688	**	24.33	LOC_Os02g37060 (photosystem II kDa protein)	10
3	RM3387	*	14.33	LOC_Os03g02020 (stress responsive A/B barrel domain-containing protein)	23
				LOC_Os03g01360 (expressed protein)	92
8	RM80	*	14.18	LOC_Os08g38300 (core histone H2A/H2B/H3/H4)	220
				LOC_Os08g39150 (expressed protein)	248
				LOC_Os08g39160 (formyl transferase)	251
				LOC_Os08g39300 (aminotransferase)	356

**, * Significant at 0.01 and 0.05 probability levels, respectively

The marker region RM6333 in chromosome 1 showed a highly significant association with the GY under drought stress. It coincides with the AI genes encoding for DNA polymerase I and receptor-like protein kinase. The contiguous marker RM11943 likewise exhibited significant association with GY under drought stress. A cluster of AI genes was found to coincide with this marker.

The markers RM5789 and RM3688 in chromosome 2 were found to coincide with the AI genes encoding for photosystem II 5 kDa and protein-binding protein. On the other hand, RM3387 at chromosome 3, which was found to align with the AI gene encoding for stress responsive A/B barrel protein (LOC_Os03g02020.2) in the co-localization analysis, is further shown here to have significant association with GY under drought stress. These findings reinforce the results obtained from the co-localization procedure.

The marker RM80 on chromosome 8 exhibited significant association with GY under drought stress. This is contiguous to the marker region RM256 (Venuprasad et al. 2009) described in the co-localization procedure. AI genes coinciding with these markers are strong candidates for further drought-yield QTL analysis.

#### Co-Localization Procedure and Selective Genotyping

Integrating the co-localization procedure and selective genotyping, allelically imbalanced genes are distributed across seven rice chromosomes – 1, 2, 3, 6, 8, 9, and 12. Two regions were confirmed using both *in silico* co-localization procedure and selective genotyping and therefore are the strongest candidates for further study on drought response. These include the regions RM3387 and RM80/RM256 in chromosomes 3 and 8, respectively. The marker region RM3387 coincides with stress responsive A/B barrel (LOC_Os03g02020); while RM80 with core histone (LOC_Os08g38300), expressed protein (LOC_Os08g39150) and formyl transferase (LOC_Os08g39160).

In summary, the ASE pipeline assayed in two contrasting conditions yielded a list of promising genes for further study. Validation of these AI genes by co-localization procedure and selective genotyping shows a strong association of these genes with drought-yield QTLs. This demonstrates that ASE method assayed on two contrasting conditions revealed the involvement of AI genes under water-stress and provided potential candidate genes for functional validation.

Our approach and findings share similarities with eQTL analysis which represents a straightforward approach in identifying candidate genes (reviewed by Druka et al. 2010). However, eQTL approach utilizes more advanced lines (e.g. recombinant inbred lines, F_3_) or double haploid whereas AI is assayed on F1 hybrids. We showed that integrating hybrid expression analysis with QTL mapping studies provides convergent evidence for potential candidate genes involved in drought response. This study opens up two avenues for further investigation: (1) test the participation of the promising candidate genes in drought response and (2) further analyze the regulatory sequences of these genes.

This paper may shed insights in assisting breeding design for hybrid crops to improve adaptation and yield to changing climate conditions. Studies using reciprocal hybrid cross is recommended for future investigation.

## Conclusions

This study reveals that AI was found inherent to rice hybrids regardless of the condition. On a genome-wide scale, water-stress conditions induce gene expression and skew the number of genes exhibiting asymmetric expression in favor of IR64-specific allele. Furthermore, changes in ASE profiles as effected by the two contrasting conditions were revealed, the most interesting of which is the bidirectional expression behavior. This happens when a gene reverses its allele preference from one condition to the other. This gives information that preferential expression (and possibly dominance) for some genes is condition-mediated as a consequence of gene-environment interactions.

The integration of two inbred genomes in the F1 may bring polymorphism in the *cis*-acting regulatory elements that causes AI since both alleles are exposed to the same pool of *trans* factors. The two inbred genomes allow the hybrid to dynamically “select” either one or both alleles, allowing more “options” to express the superior alleles which may provide the hybrids a wider expression and subsequently phenotypic advantages.

We then validated the involvement of these AI genes using *in-silico* co-localization analysis and selective genotyping. Our results showed a strong association of the genes asymmetrically expressed with previously published drought-QTL markers and markers selected in this study for selective genotyping. This suggests that important trait-associated genes are asymmetrically expressed in the F1 and therefore AI assayed in the hybrid may be an efficient approach to determine candidate genes associated with the phenotype.

## Methods

### Dry-Down Experiment

In this study, rice (*Oryza sativa* L.) F1 hybrids were obtained by crossing IR64 and Apo to study AI under normal and drought conditions. Seeds from the parents and the hybrids were grown in petri plates and were transferred to small pots after germination. Leaf samples from the seedlings were collected to test the hybrid status of the plants. Genomic DNA was extracted using the modified CTAB method as described by Murray and Thompson (1980). Hybrids were confirmed using SSR markers RM 269, RM 511, and RM 80. After hybrid confirmation, seedlings were transplanted in large pots. The parental and hybrid plants were adequately fertilized and grown under controlled conditions in the phytotron, at IRRI. For water-limiting condition, the fraction of transpirable soil water (FTSW) dry-down approach was used as previously described (Cal et al. 2013; Serraj et al. 2014; Sinclair and Ludlow 1986). Water-limiting condition was imposed on stress-treated plants by initiating a soil dry-down protocol starting 10 days before heading until the plants reached 0.5 FTSW.

All pots were weighed daily to calculate the amount of water lost. All genotypes were replicated four times (IR64, Apo and their F1), for each of the two treatments (control and 0.5 FTSW), giving a total of 24 samples, randomly arranged in the phytotron.

### RNA Extraction

Leaf samples from each plant were collected at the end of the dry-down treatment. RNA was extracted using the TRIzol method according to the instructions provided by the supplier (Invitrogen, San Diego, Calif., USA). RNA samples were treated with DNAse to remove DNA contamination. RNA concentrations were estimated using a NanoDrop 1000 Spectrophotometer (NanoDrop Technologies, Wilmington, Del., USA).

RNA-seq libraries were made as described in Illumina’s standard protocol for RNA-seq using the parental (IR64 and Apo) and F1 RNA samples from each treatment (normal and water-stress). Libraries were sequenced on Illumina GAIIx, generating 38- and 90-base paired end (PE) reads, for the first and second sequencing protocols, respectively. Two biological replicates were sent for sequencing to satisfy minimum requirements for RNAseq as stipulated in the Standards, Guidelines and Best Practices of the ENCODE Consortium (http://genome.ucsc.edu/). Raw reads were received in fastq format.

### Data Processing and Analysis

#### Quality Checking and Trimming

Quality filtering of PE reads was performed using FASTQ Quality Filter. Filtered reads were trimmed off by FASTQ Quality Trimmer from FASTX-Toolkit (http://hannonlab.cshl.edu/). We checked the 75 % percentile of the sequencing qualities for each base of the PE reads in each replicate. Bases with 75 % percentile of the sequencing qualities < 28 were removed for all reads in the replicate. All commands are described in the Additional file 13: S1.

#### Mapping and Generating Consensus Pseudo-Reference

After read trimming, a pseudo-reference was generated by mapping Apo and IR64 reads separately against the indexed reference sequence *O. sativa* ssp. *japonica* (cv. Nipponbare) MSU v7 cDNA (http://rice.plantbiology.msu.edu/) using bowtie2. Because of the variations between *indica* and *japonica* (Nipponbare reference genome), alignment procedure was set at lenient parameters to allow more significant mapping using the ‘score function’.

#### Post-Processing

After mapping, we used samtools sort and mpileup commands (Li and Durbin 2009) to parse the mapping results. PERL scripts were written to call SNPs and InDels common to Apo and IR64 sequences but different from the Nipponbare reference (see Additional file 14: Figure S3 for schematic diagram). SNPs and InDels were called between the *indica* genotypes and the Nipponbare reference genome, a *japonica*. SNP reads must have at least 5× coverage and SNP proportion must be more than 0.8. For InDels, read coverage must also be at least 5× but InDel proportion should be at least 0.5.

The consensus pseudo-reference was generated by (1) replacing the SNPs common to Apo and IR64 but not in the MSU v7 cDNA, and (2) incorporating the common InDels found in both Apo and IR64 (Additional file 14: Figure S3).

The pseudo-reference was mapped against the 93-11 *indica* RNAseq reads downloaded from BGI (http://rise2.genomics.org.cn/page/rice/index.jsp). We mapped the 93-11 reads (*indica*) against the Nipponbare. This is to assess any increase in alignment among the reads. *Indica-indica* alignment (i.e., 93-11 and our pseudo-reference) should be higher than *indica*-*japonica* (i.e., 93-11: Nipponbare reference genome).

### SNP Calling

Bowtie2 was used to map Apo, IR64 and F1 to the pseudo-reference. Samtools sort and mpileup commands were then used to parse the mapping results. A PERL script was used to find the SNPs between Apo and IR64 sequences. SNP reads must have at least 3× read coverage and SNP proportion must be more than 0.8 (Additional file 15 Figure S4).

### Identifying the F1 Reads by Read-Wise Approach

To identify F1 reads, two approaches are possible: the SNP- and Read-wise approaches (Additional file 16 Figure S5). SNP-wise approach entails the counting of the number of SNPs from each parent-specific allele. A schematic diagram is shown in Additional file 16 Figure S5 to illustrate this strategy. The number of SNPs corresponding to Apo were 10; IR64, 6. Apparently, SNP-wise approach tends to overestimate the read counts.

Read-wise approach, on the other hand, uses SNPs between the parents to guide us in identifying which genotype-specific reads belongs to which parental genotype in the hybrid. The number of reads corresponding to Apo were 8; IR64, 4. We preferred the Read- over SNP-wise approach as it gives more accurate number of read counts. This approach uses SNPs between the two parent-specific alleles as copy-specific tags. These variants allow us to distinguish and quantify the two SNP alleles in the heterozygote. A PERL script was used to carry out this strategy.

#### Parental Reads Extraction and Gene Expression Estimation

Using the mapping results of Apo and IR64 for the pseudo-reference sequence (bam files) and SNP information, we extracted the parental reads containing SNP by a PERL script. We used eXpress (Roberts and Pachter 2013) to perform gene expression analysis. We then counted the reads to estimate gene expression level from mapping results of F1 to the pseudo-reference. We identified genotype-specific reads guided by the parent-specific SNPs in the F1.

#### Differences in Gene Expression Analysis Using DESeq

Allele-specific expression levels were estimated in the hybrids between the two treatments in both replicates using DESeq. Before testing, we normalized samples by dividing the corresponding size factor to minimize the difference between samples. We tested the significance of differential expression in the hybrids.

#### AI test

Binomial test was employed to test significance of AI between the two alleles in the hybrid. We used DESeq package in R (Anders and Huber 2010) to calculate the size factors for normalization, then we normalized the expression values by dividing the corresponding size factors. The normalized expression of the two replicates were combined and tested for significance of imbalance using binomial and chi square tests.

To satisfy sample size requirement of normal approximation by chi-square test, genes with expected values less than 5 were removed. Gramene (www.gramene.org) was used to find the annotation and biological function of genes.

#### Calculating FC and Determining Condition-Mediated ASE Profiles of Genes

After normalization and read count adjustments, expression ratios and log_2_FC were calculated. Expression ratios were obtained by dividing the transcript expression of Apo over IR64 (Apo/IR64) then were log-transformed. To infer condition-mediated ASE changes of genes, we pairwise-compared their log_2_FCs under non- and water-stress conditions (Additional file 1: Table S1 and Additional file 2: Table S2). We only considered genes with *P* values < 0.05 in one or both conditions. Using this lenient strategy, 976 genes were shortlisted for this analysis (Additional file 7: Table S5). If we impose a more stringent strategy (i.e. *P* < 0.05 in both conditions except for tPAV genes which are expressed in only one condition), only 351 genes are shortlisted (data not shown). We preferred the lenient approach to obtain more information on expression profiles and include a wider number of genes. Asymmetrically expressed genes (biallelic) have expression values lower than the cutoff of 1.25× fold. Their condition-mediated ASE behaviors cannot be ascertained. Both alleles are expressed relatively equal and therefore cannot be classified as Apo- or IR64-specific allele preferring.

### Association Analysis of AI Genes with Drought-Response QTL

#### *In-Silico* Association

To initially confirm putative involvement with grain yield (GY) under drought condition, asymmetrically expressed genes were aligned with the markers previously identified (Venuprasad et al. 2009). These molecular marker loci showed highly significant allele frequency differences between stress-selected (SS) and non-stress selected (NS) sets of F_2:3_ lines derived from Apo/IR64 cross. This approach entails anchoring the positions of the SSR markers and AI genes to estimate their physico-genetic linkage on Gramene using *O. sativa indica* (www.gramene.org).

#### Selective Genotyping

After the alignment procedure, the involvement of the AI genes with drought tolerance was tested by selective genotyping. Forty (20 highest and 20 lowest yielding lines from each of the tails of the whole population) of 160 Apo × IR64 F_3:5_ RILs were genotyped using 153 SSR markers residing closely to or co-localizing with the genes.

#### Phenotypic Data Collection

The RILs were screened under lowland drought stress in dry season in 2009 at the experiment station of the International Rice Research Institute (IRRI), Los Baños, Laguna, Philippines.

Trial management, population generation and phenotypic data collection of progenies comprising of 160 lines were performed using the protocol outlined under lowland conditions. All the trials were laid out as alpha lattice designs, with plot length of 5.25 m, and spacing between rows was 0.20 m under drought stress. Twenty-one-day-old seedlings were transplanted then stress was applied after 28 days. The number of rows per plot was two; two replications were used in all trials.

From the upland (stress) trial of DS 2009, phenotypic data on grain yield were collected. Least square (LS) means was computed between two replicates of each line for all phenotypic data.

#### Genotyping

All molecular marker work was conducted at the Molecular Genetics Laboratory, Genetics and Biotechnology (GB) Division, IRRI. Leaf samples of the 160 lines were collected from Field 202, IRRI experiment station. Samples were placed in -20 °C freezer until extraction. Miniprep scale DNA extraction was performed using a modified CTAB protocol by Sambrook and Russell (2001). The concentration of DNA was analyzed using NanoDrop® ND-1000 Spectrophotometer and adjusted to ~50 ηg/μL.

A parental polymorphism survey was carried out using 153 SSR markers between Apo and IR64 parents. Only 22 (14 %) of the markers exhibited clear polymorphism and were used to genotype the lines identified to belong to the two tail ends.

PCR amplification of all markers was performed in 20 μL reactions containing 2 μL 10 × buffer, 1.5 μL 25 mM MgCl_2_, 1.6 μL 2 mM dNTPs, 1 μL 5 μM forward and reverse primers and 0.5 μL of Taq DNA polymerase (4U/ μL) and 10-50 ηg μL-1 gDNA using 96-well thermal cycler. After initial denaturation for 5 min at 94 °C, each cycle comprised 1 min denaturation at 94 °C, 2 min annealing at 50-65 °C (depending on the primer pair), and 2 min extension at 72 °C with a final extension for 5 min at the end of 35 cycles. PCR products were mixed with bromophenol blue loading dye and were analyzed by PAGE (8 % gel solution) for 1.5 h, 100 V, 100 mA. Gels were stained with SYBR Safe and viewed using GelDoc System.

The two tails of the RILs were scored according to the parental banding pattern as IR64 homozygotes (1), Apo homozygotes (2) or heterozygotes (3). When no banding pattern or non-parental bands were observed, they were treated as missing values (0).

Association analysis between the genotypic and phenotypic data was performed using Single Marker Regression Analysis. All rice microsatellite (RM) markers described in this paper were taken from Gramene (http://www.gramene.org/) as described by Temnykh et al. (2001).

## Additional Files

Additional file 1: Table S1. List of genes in the hybrids with SNPs between the two parent-specific alleles under normal conditions. (XLSX 233 kb)
Additional file 2: Table S2. List of genes in the hybrids with SNPs between the two parent-specific alleles under water-stress conditions. (XLSX 220 kb)
Additional file 3: Table S3. List of genes in the hybrids exhibiting significant DE between the two parent-specific alleles at *P* < 0.05 under normal conditions. (XLSX 81 kb)
Additional file 4: Table S4. List of genes in the hybrids exhibiting significant DE between the two parent-specific alleles at *P* < 0.05 under water-stress conditions. (XLSX 111 kb)
Additional file 5: Figure S1A. Heatmaps showing genes in the F1 hybrid exhibiting ASE under normal conditions. Green cells represent values when IR64 allele is preferred; red, when Apo is preferred in the F1 hybrid. The black cells represent a symmetrical (or equal) expression between the two genotype-specific alleles. Color-scale bar is also shown. (PNG 895 kb)
Additional file 6: Figure S1B. Heatmaps showing genes in the F1 hybrid exhibiting ASE under water-stress conditions. Description is similar to Additional file 5: Figure S1A. (PNG 805 kb)
Additional file 7: Table S5. Pairwise comparison of the expression preference of the hybrid genes exposed under normal and water-stress conditions. It shows the condition-mediated changes in expression preference of each gene. (XLSX 155 kb)
Additional file 8: Figure S2A. Heatmaps of genes between non- (Column 1) and water-stress (Column 2) conditions exhibiting tPAV. Genes are induced during non-stress conditions (tPAVnormal). The first column represents the ASE preference of a gene under normal conditions (Control) and the second under stress conditions (Stress). Descriptions of color and color scale bar are similar to Additional file 5: Figure S1A. (JPG 182 kb)
Additional file 9: Figure S2B. Heatmaps of genes between non- (Column 1) and water-stress (Column 2) conditions exhibiting tPAV. Genes are induced during water-stress conditions (tPAVstress). Descriptions of color and color scale bar are similar to Additional file 5: Figure S1A and Additional file 8: Figure S2A. (JPG 117 kb)
Additional file 10: Figure S2C. Heatmaps of genes between non- (Column 1) and water-stress conditions (Column 2) exhibiting bidirectional expression behavior. Green (Column 1; Control) to red cells (column 2; Stress) indicate genes with preferential expression changes from IR64- to Apo-specific allele. Red (Column 1; Control) to green cells (column 2; Stress) indicate genes with preferential expression changes from Apo- to IR64-specific allele. Description of colors and color scale bar is similar to Additional file 5: Figure S1A and Additional file 8: Figure S2A. (JPG 311 kb)
Additional file 11: Figure S2D. Heatmaps of genes between non- (Column 1) and water-stress conditions (Column 2) exhibiting unidirectional expression behavior (IR64-favoring genes). Green (Column 1; Control) to green cells (column 2; Stress) indicate genes which consistently prefer the IR64-specific allele regardless of the conditions. Description of colors and color scale bar is similar to Additional file 5: Figure S1A and Additional file 8: Figure S2A. (JPG 1096 kb)
Additional file 12: Figure S2E. Heatmaps of genes between non- (Column 1) and water-stress conditions (Column 2) exhibiting unidirectional expression behaviour (Apo-favor genes). Red (Column 1; Control) to red cells (column 2; Stress) indicate genes which consistently prefer the Apo-specific allele regardless of the conditions. Description of colors and color scale bar is similar to Additional file 5: Figure S1A and Additional file 8: Figure S2A. (JPG 1029 kb)
Additional file 13: S1. Commands used for AI analysis. (DOCX 15 kb)
Additional file 14: Figure S3. Schematic diagram illustrating our method of generating a pseudo-reference sequence. Bases common to Apo and IR64 but different from the Nipponbare reference genome were called. These variations were used to generate the pseudo-reference. (JPG 58 kb)
Additional file 15: Figure S4. A schematic representation to call SNPs. By using the SNPs between the parents, we can use the information to determine the expression of alleles in the F1 hybrids. In the figure, Apo and IR64 have two SNP loci. In the first locus the read coverage is more than 3 for Apo and most of the genotypes are T (more than 80 %) so this locus is defined as ‘T’ SNP. Compared to Apo, IR64 has a read coverage of more than 3 and most of the genotypes are C (more than 80 %) so this locus is defined as ‘C’ SNP for IR64. (JPG 33 kb)
Additional file 16: Figure S5. Schematic diagram illustrating Read- and SNP-wise approaches to identify genotype-specific alleles in F1. Using SNPs between the parents as copy-sequence tags, genotype-specific alleles in the hybrid can be identified. (JPG 44 kb)

## Abbreviations

AI: Allelic imbalance
ASE: Allele-specific expression
DE: Differential expression
FTSW: Fraction of transpirable soil water
GY: Grain yield
NAM: No apical meristem
QTL: Quantitative trait loci
SNP: Single nucleotide polymorphism
TF: Transcription factors.
