# Supplementary figures and images for: Analysis of Allelic Imbalance in Rice Hybrids Under Water Stress and Association of Asymmetrically Expressed Genes with Drought-Response QTLs
Source: Rice (N Y). 2016 Sep 26;9:50. doi: 10.1186/s12284-016-0123-4 (PMC5037104; doi:10.1186/s12284-016-0123-4)

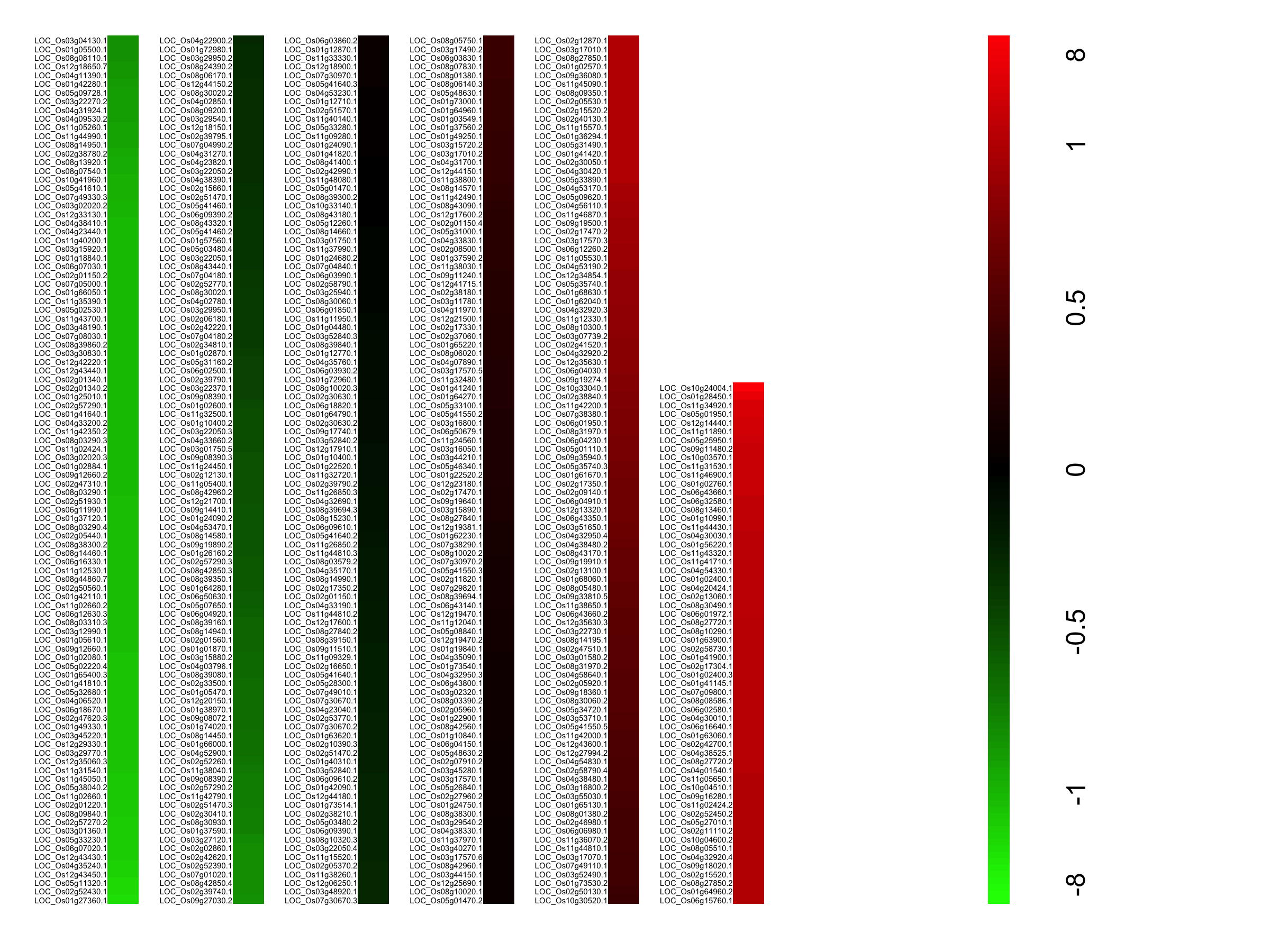

Supplement: Additional file 5: Figure S1A. — Heatmaps showing genes in the F1 hybrid exhibiting ASE under normal conditions. Green cells represent values when IR64 allele is preferred; red, when Apo is preferred in the F1 hybrid. The black cells represent a symmetrical (or equal) expression between the two genotype-specific alleles. Color-scale bar is also shown. (PNG 895 kb) [file 12284_2016_123_MOESM5_ESM.png]

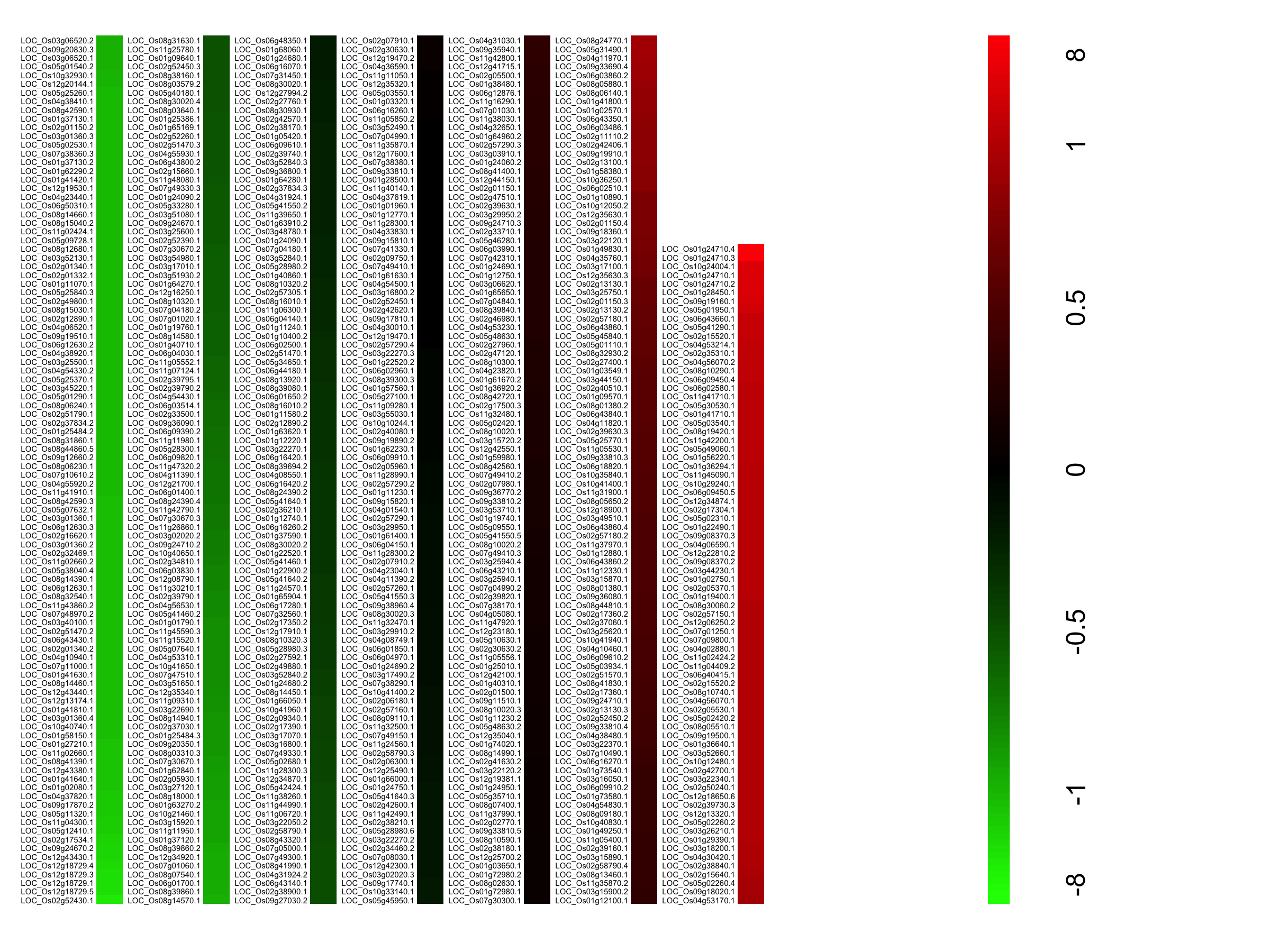

Supplement: Additional file 6: Figure S1B. — Heatmaps showing genes in the F1 hybrid exhibiting ASE under water-stress conditions. Description is similar to Additional file 5: Figure S1A. (PNG 805 kb) [file 12284_2016_123_MOESM6_ESM.png]

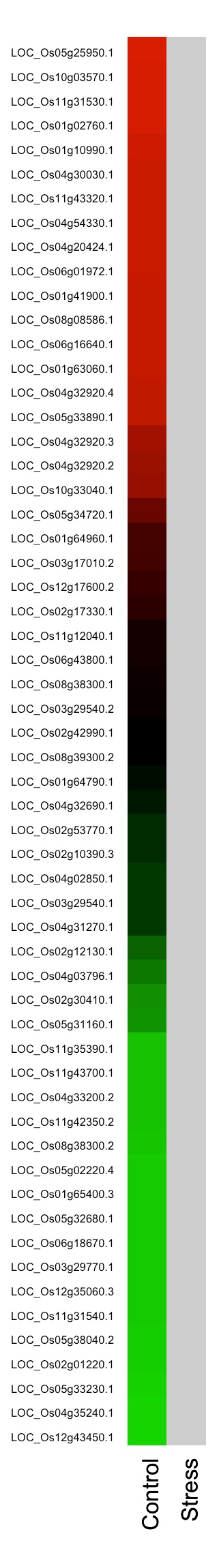

Supplement: Additional file 8: Figure S2A. — Heatmaps of genes between non- (Column 1) and water-stress (Column 2) conditions exhibiting tPAV. Genes are induced during non-stress conditions (tPAVnormal). The first column represents the ASE preference of a gene under normal conditions (Control) and the second under stress conditions (Stress). Descriptions of color and color scale bar are similar to Additional file 5: Figure S1A. (JPG 182 kb) [file 12284_2016_123_MOESM8_ESM.jpg]

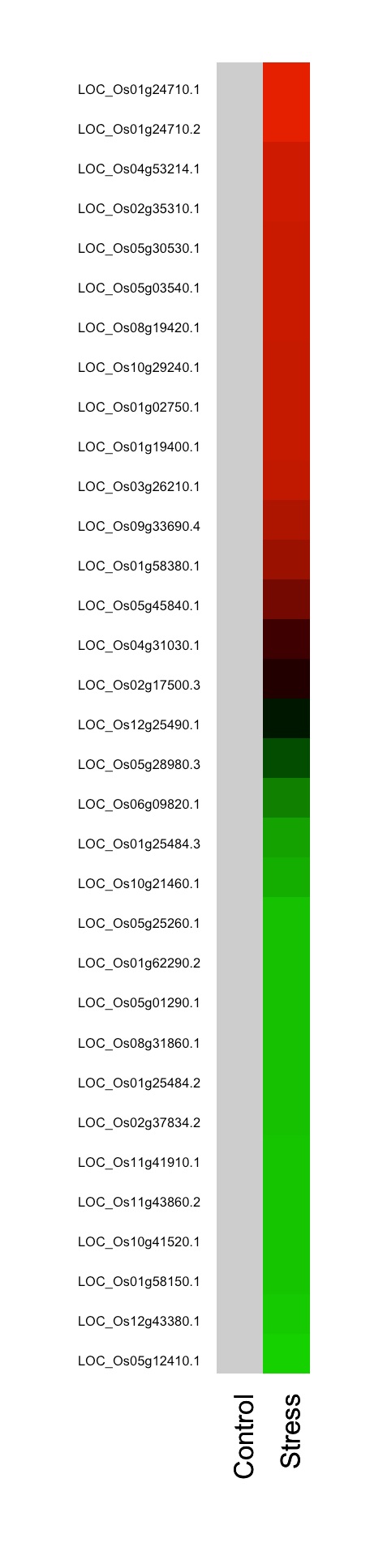

Supplement: Additional file 9: Figure S2B. — Heatmaps of genes between non- (Column 1) and water-stress (Column 2) conditions exhibiting tPAV. Genes are induced during water-stress conditions (tPAVstress). Descriptions of color and color scale bar are similar to Additional file 5: Figure S1A and Additional file 8: Figure S2A. (JPG 117 kb) [file 12284_2016_123_MOESM9_ESM.jpg]

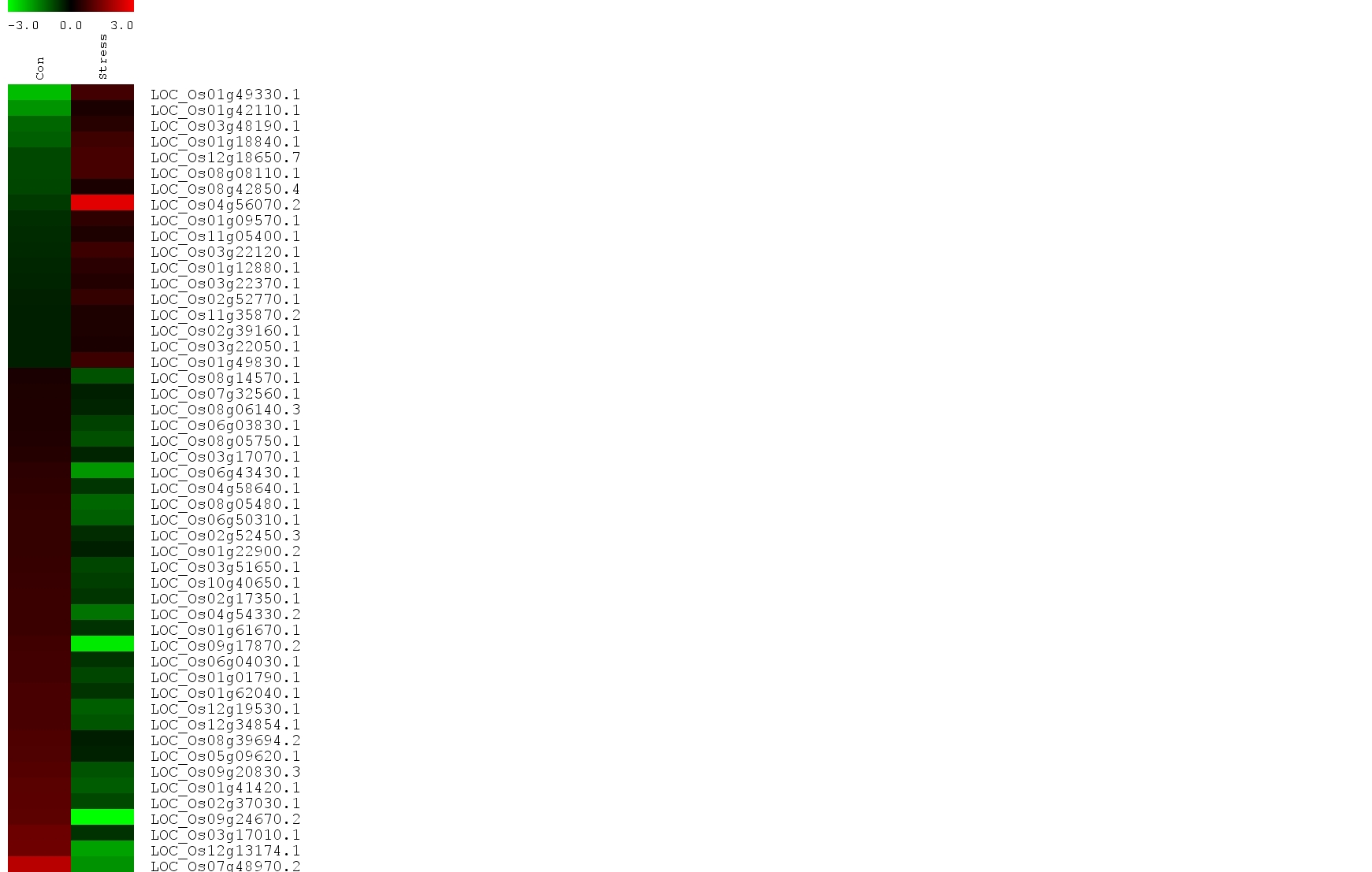

Supplement: Additional file 10: Figure S2C. — Heatmaps of genes between non- (Column 1) and water-stress conditions (Column 2) exhibiting bidirectional expression behavior. Green (Column 1; Control) to red cells (column 2; Stress) indicate genes with preferential expression changes from IR64- to Apo-specific allele. Red (Column 1; Control) to green cells (column 2; Stress) indicate genes with preferential expression changes from Apo- to IR64-specific allele. Description of colors and color scale bar is similar to Additional file 5: Figure S1A and Additional file 8: Figure S2A. (JPG 311 kb) [file 12284_2016_123_MOESM10_ESM.jpg]

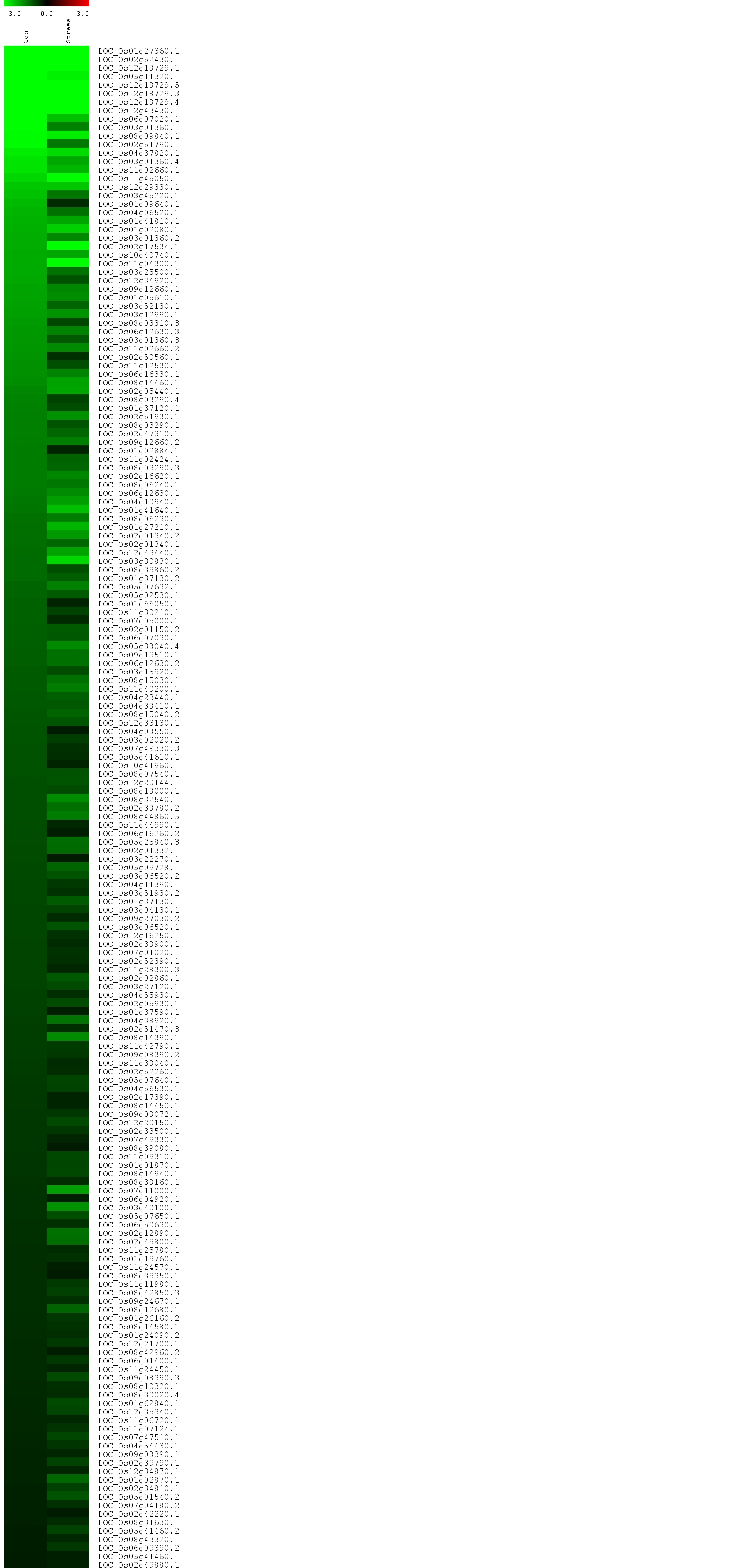

Supplement: Additional file 11: Figure S2D. — Heatmaps of genes between non- (Column 1) and water-stress conditions (Column 2) exhibiting unidirectional expression behavior (IR64-favoring genes). Green (Column 1; Control) to green cells (column 2; Stress) indicate genes which consistently prefer the IR64-specific allele regardless of the conditions. Description of colors and color scale bar is similar to Additional file 5: Figure S1A and Additional file 8: Figure S2A. (JPG 1096 kb) [file 12284_2016_123_MOESM11_ESM.jpg]

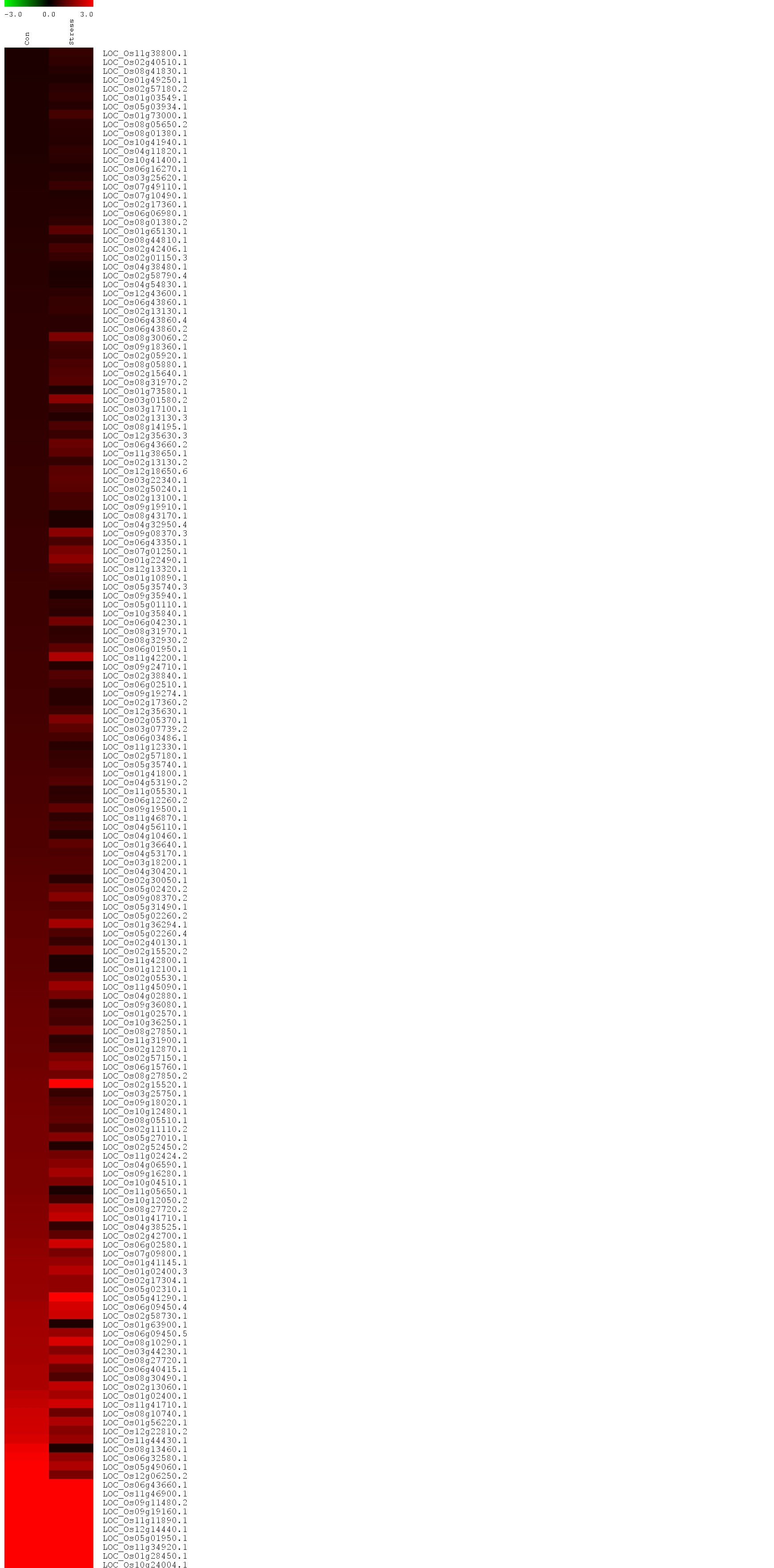

Supplement: Additional file 12: Figure S2E. — Heatmaps of genes between non- (Column 1) and water-stress conditions (Column 2) exhibiting unidirectional expression behaviour (Apo-favor genes). Red (Column 1; Control) to red cells (column 2; Stress) indicate genes which consistently prefer the Apo-specific allele regardless of the conditions. Description of colors and color scale bar is similar to Additional file 5: Figure S1A and Additional file 8: Figure S2A. (JPG 1029 kb) [file 12284_2016_123_MOESM12_ESM.jpg]

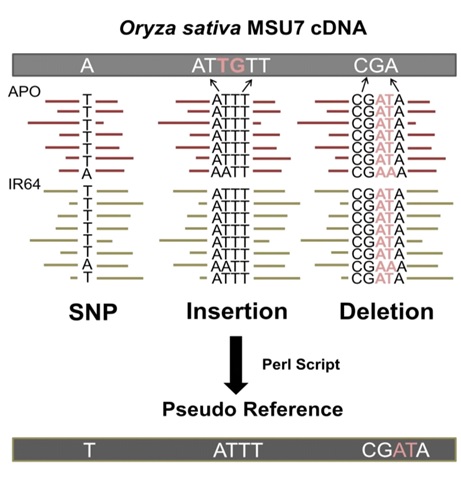

Supplement: Additional file 14: Figure S3. — Schematic diagram illustrating our method of generating a pseudo-reference sequence. Bases common to Apo and IR64 but different from the Nipponbare reference genome were called. These variations were used to generate the pseudo-reference. (JPG 58 kb) [file 12284_2016_123_MOESM14_ESM.jpg]

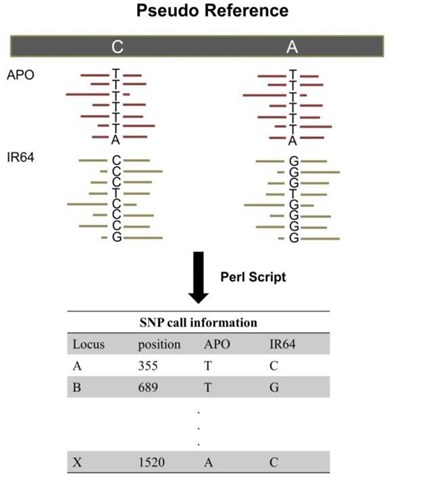

Supplement: Additional file 15: Figure S4. — A schematic representation to call SNPs. By using the SNPs between the parents, we can use the information to determine the expression of alleles in the F1 hybrids. In the figure, Apo and IR64 have two SNP loci. In the first locus the read coverage is more than 3 for Apo and most of the genotypes are T (more than 80 %) so this locus is defined as ‘T’ SNP. Compared to Apo, IR64 has a read coverage of more than 3 and most of the genotypes are C (more than 80 %) so this locus is defined as ‘C’ SNP for IR64. (JPG 33 kb) [file 12284_2016_123_MOESM15_ESM.jpg]

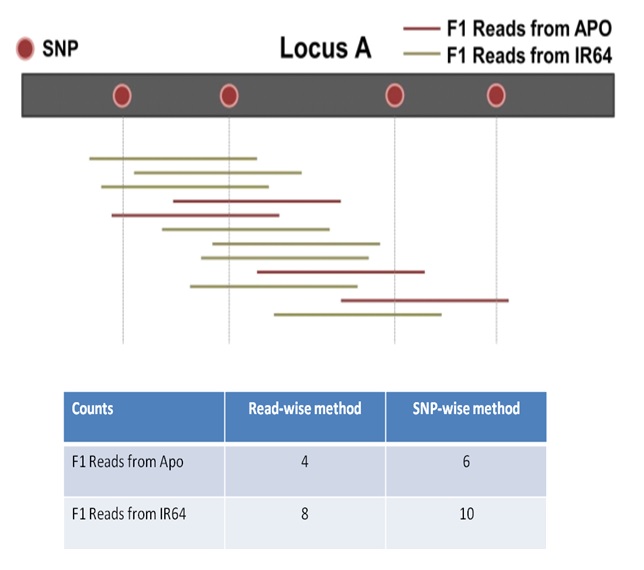

Supplement: Additional file 16: Figure S5. — Schematic diagram illustrating Read- and SNP-wise approaches to identify genotype-specific alleles in F1. Using SNPs between the parents as copy-sequence tags, genotype-specific alleles in the hybrid can be identified. (JPG 44 kb) [file 12284_2016_123_MOESM16_ESM.jpg]
