# Supplementary material for: Analysis of Allelic Imbalance in Rice Hybrids Under Water Stress and Association of Asymmetrically Expressed Genes with Drought-Response QTLs
Source: Rice (N Y). 2016 Sep 26;9:50. doi: 10.1186/s12284-016-0123-4 (PMC5037104; doi:10.1186/s12284-016-0123-4)
Supplement: Additional file 13: S1. — Commands used for AI analysis. (DOCX 15 kb) [file 12284_2016_123_MOESM13_ESM.docx]

The following scripts make up our pipeline to determine DE between genotypes and ASE imbalance in the F1 (see Materials and Method).

1. data:

Rep1: (ir64, apo, f1)x(control, stress) bp:38 Q33(quality score type)

Rep2: (ir64, apo, f1)x(control, stress) bp:90 Q64(quality score type)

##name of fastq file

Rep1_APO-C_1.fq Rep1_APO-C_2.fq

Rep1_APO-S_1.fq Rep1_APO-S_2.fq

Rep1_IR64-C_1.fq Rep1_IR64-C_2.fq

Rep1_IR64-S_1.fq Rep1_IR64-S_2.fq

Rep1_F1-C_1.fq Rep1_F1-C_2.fq

Rep1_F1-S_1.fq Rep1_F1-S_2.fq

Rep2_APO-C_1.fq Rep2_APO-C_2.fq

Rep2_APO-S_1.fq Rep2_APO-S_2.fq

Rep2_IR64-C_1.fq Rep2_IR64-C_2.fq

Rep2_IR64-S_1.fq Rep2_IR64-S_2.fq

Rep2_F1-C_1.fq Rep2_F1-C_2.fq

Rep2_F1-S_1.fq Rep2_F1-S_2.fq

2. Quality control

2-1. FastQC

download:http://www.bioinformatics.babraham.ac.uk/projects/fastqc/fastqc_v0.10.1.zip

install README: http://www.bioinformatics.babraham.ac.uk/projects/fastqc/INSTALL.txt

#!/bin/bash

for i in APO IR64 F1;do

fastqc -f fastq Rep1_${i}-C_1.fq Rep1_${i}-C_2.fq &

fastqc -f fastq Rep1_${i}-S_1.fq Rep1_${i}-S_2.fq &

fastqc -f fastq Rep2_${i}-C_1.fq Rep2_${i}-C_2.fq &

fastqc -f fastq Rep2_${i}-S_1.fq Rep2_${i}-S_2.fq &

done

2-2. FASTX Toolkit

http://hannonlab.cshl.edu/fastx_toolkit/

#!/bin/bash

for i in APO IR64 F1;do

fastx_trimmer -t 10 -m 75 -v -i ../Raw_reads/Replicate2/Rep2_${i}-C_1.fq -o QC_Rep2_${i}-C_1.fq &

fastx_trimmer -t 10 -m 75 -v -i ../Raw_reads/Replicate2/Rep2_${i}-C_2.fq -o QC_Rep2_${i}-C_2.fq &

done

3. Mapping Method: Pseudo Reference

3-1. download reference

wget ftp://ftp.plantbiology.msu.edu/pub/data/Eukaryotic_Projects/o_sativa/annotation_dbs/pseudomolecules/version_7.0/all.dir/all.cdna

mv all.cdna MSU7_all.cdna (change file name)

3-2.bowtie2 index

bowtie2-build -f MSU7_all.cdna MSU7

3-3.bowtie2 mapping

Replicate 1:

#!/bin/bash

for i in APO IR64 F1;do

bowtie2 --rdg 6,5 --rfg 6,5 --score-min L,-.7,-.7 --mp 5,2 -X 500 --no-mixed --no-discordant --fr -x MSU7 -q --phred33 -p 5 -1 ../FastQC/Rep1_${i}-C_1.fq -2 ../FastQC/Rep1_${i}-C_2.fq | samtools view -bS - -o Rep1_${i}-C.bam

bowtie2 --rdg 6,5 --rfg 6,5 --score-min L,-.7,-.7 --mp 5,2 -X 500 --no-mixed --no-discordant --fr -x MSU7 -q --phred33 -p 5 -1 ../FastQC/Rep1_${i}-S_1.fq -2 ../FastQC/Rep1_${i}-S_2.fq | samtools view -bS - -o Rep1_${i}-S.bam

done

Replicate 2:

#!/bin/bash

for i in APO IR64 F1;do

bowtie2 --rdg 6,5 --rfg 6,5 --score-min L,-.7,-.7 --mp 5,2 -X 600 --no-mixed --no-discordant --fr -x MSU7 -q --phred64 -p 5 -1 ../FastQC/QC_Rep2_${i}-C_1.fq -2 ../FastQC/QC_Rep2_${i}-C_2.fq | samtools view -bS - -o Rep2_${i}-C.bam

bowtie2 --rdg 6,5 --rfg 6,5 --score-min L,-.7,-.7 --mp 5,2 -X 600 --no-mixed --no-discordant --fr -x MSU7 -q --phred64 -p 5 -1 ../FastQC/QC_Rep2_${i}-S_1.fq -2 ../FastQC/QC_Rep2_${i}-S_2.fq | samtools view -bS - -o Rep2_${i}-S.bam

done

3-4. Samtools sort

Replicate 1:

#!/bin/bash

for i in APO IR64 F1;do

samtools sort Rep1_${i}-S.bam Rep1_${i}-S_sorted &

samtools sort Rep1_${i}-C.bam Rep1_${i}-C_sorted &

done

Replicate 2:

#!/bin/bash

for i in APO IR64 F1;do

samtools sort Rep2_${i}-S.bam Rep2_${i}-S_sorted &

samtools sort Rep2_${i}-C.bam Rep2_${i}-C_sorted &

done

3-5. Samtools mpileup

#!/bin/bash

samtools mpileup -I -f MSU7_all.cdna Rep1_APO-C_sorted.bam Rep1_APO-S_sorted.bam Rep1_IR64-C_sorted.bam Rep1_IR64-S_sorted.bam > Rep1_pool_mpileup.txt &

samtools mpileup -I -f MSU7_all.cdna Rep2_APO-C_sorted.bam Rep2_APO-S_sorted.bam Rep2_IR64-C_sorted.bam Rep2_IR64-S_sorted.bam > Rep2_pool_mpileup.txt &

#samtools mpileup -I -f MSU7_all.cdna Rep1_F1-C_sorted.bam Rep1_F1-S_sorted.bam > Rep1_f1_mpileup.txt &

#samtools mpileup -I -f MSU7_all.cdna Rep2_F1-C_sorted.bam Rep2_F1-S_sorted.bam > Rep2_f1_mpileup.txt &

3-6. modify reference

Commonbase call:

perl commonbase_checkok.pl Rep1_pool_mpileup.txt > Rep1_common_basecall.txt

perl commonbase_checkok.pl Rep2_pool_mpileup.txt > Rep2_common_basecall.txt

cat Rep1_common_basecall.txt Rep2_common_basecall.txt | sort | uniq > all_rep_commonbase.txt

perl commonbase_f1_checkok.pl Rep1_f1_mpileup.txt > Rep1_f1_commonbase.txt

perl commonbase_f1_checkok.pl Rep2_f1_mpileup.txt > Rep2_f1_commonbase.txt

cat Rep1_f1_commonbase.txt Rep2_f1_commonbase.txt | sort | uniq > all_pool_f1_commonbase.txt

cat all_rep_commonbase_2.txt all_pool_f1_commonbase.txt | sort | uniq > all_pool_commonbase.txt

InDel call:

perl indel_checkok.pl Rep1_pool_mpileup.txt > Rep1_indel.txt

perl indel_checkok.pl Rep2_pool_mpileup.txt > Rep2_indel.txt

cat Rep1_indel.txt Rep2_indel.txt | sort | uniq > all_rep_indel.txt

perl indel_f1_checkok.pl Rep1_f1_mpileup.txt > Rep1_f1_indel.txt

perl indel_f1_checkok.pl Rep2_f1_mpileup.txt > Rep1_f2_indel.txt

cat Rep1_f1_indel.txt Rep2_f1_indel.txt | sort | uniq > all_pool_f1_indel.txt

cat all_rep_indel.txt all_pool_f1_indel.txt | sort | uniq > all_pool_indel.txt

Modify Reference:

perl reference_commonbaseModify_checkok.pl MSU7_all.cdna all_pool_commonbase.txt > mod_commonbase.fasta

perl reference_indelModify_checkok.pl mod_commonbase.fasta all_pool_indel.txt > modwithindel_ref.fasta

3-7 Pseudo Reference check

bowtie2 -x MSU7 -q --phred33 -p 12 -1 test_1.fq -2 test_2.fq | samtools view -bS - -o test.bam

4. Mapping Method: SNP call

4-1. bowtie2 index & samtools index

bowtie2-build -f modwithindel_ref.fasta mod_indel_MSU7

samtools faidx modwithindel_ref.fasta

4-2. bowtie2 mapping

Replicate 1:

#!/bin/bash

for i in APO IR64 F1;do

bowtie2 --rdg 6,5 --rfg 6,5 --score-min L,-.6,-.4 -X 500 --no-mixed --no-discordant --fr -x mod_indel_MSU7 -q --phred33 -p 5 -1 ../FastQC/Rep1_${i}-C_1.fq -2 ../FastQC/Rep1_${i}-C_2.fq | samtools view -bS - -o mod_Rep1_${i}-C.bam

bowtie2 --rdg 6,5 --rfg 6,5 --score-min L,-.6,-.4 -X 500 --no-mixed --no-discordant --fr -x mod_indel_MSU7 -q --phred33 -p 5 -1 ../FastQC/Rep1_${i}-S_1.fq -2 ../FastQC/Rep1_${i}-S_2.fq | samtools view -bS - -o mod_Rep1_${i}-S.bam

done

Replicate 2:

#!/bin/bash

for i in APO IR64 F1;do

bowtie2 --rdg 6,5 --rfg 6,5 --score-min L,-.6,-.4 -X 600 --no-mixed --no-discordant --fr -x mod_indel_MSU7 -q --phred64 -p 5 -1 ../FastQC/QC_Rep2_${i}-C_1.fq -2 ../FastQC/QC_Rep2_${i}-C_2.fq | samtools view -bS - -o mod_Rep2_${i}-C.bam

bowtie2 --rdg 6,5 --rfg 6,5 --score-min L,-.6,-.4 -X 600 --no-mixed --no-discordant --fr -x mod_indel_MSU7 -q --phred64 -p 5 -1 ../FastQC/QC_Rep2_${i}-S_1.fq -2 ../FastQC/QC_Rep2_${i}-S_2.fq | samtools view -bS - -o mod_Rep2_${i}-S.bam

done

4-3. Samtools sort

#!/bin/bash

for i in APO IR64;do

samtools sort mod_Rep1_${i}-S.bam mod_Rep1_${i}-S_sorted &

samtools sort mod_Rep1_${i}-C.bam mod_Rep1_${i}-C_sorted &

done

for i in APO IR64;do

samtools sort mod_Rep2_${i}-S.bam mod_Rep2_${i}-S_sorted &

samtools sort mod_Rep2_${i}-C.bam mod_Rep2_${i}-C_sorted &

done

4-4. Samtools mpileup

#!/bin/bash

samtools mpileup -I -f modwithindel_ref.fasta mod_Rep1_APO-C_sorted.bam mod_Rep1_APO-S_sorted.bam mod_Rep1_IR64-C_sorted.bam mod_Rep1_IR64-S_sorted.bam > mod_Rep1_pool_mpileup.txt &

samtools mpileup -I -f modwithindel_ref.fasta mod_Rep2_APO-C_sorted.bam mod_Rep2_APO-S_sorted.bam mod_Rep2_IR64-C_sorted.bam mod_Rep2_IR64-S_sorted.bam > mod_Rep2_pool_mpileup.txt &

4-5. SNP call

perl snpcall_checkok.pl mod_Rep1_pool_mpileup.txt > Rep1_pool_snpcall.txt

perl snpcall_checkok.pl mod_Rep2_pool_mpileup.txt > Rep2_pool_snpcall.txt

cat Rep1_pool_snpcall.txt Rep2_pool_snpcall.txt | sort | uniq > all_rep_snpcall.txt

5. Extract F1 read count & Extract Parent read count

5-1. extract F1 reac count

perl new_extract_f1_1c.pl mod_Rep1_F1-C.bam all_rep_snpcall.txt &

perl new_extract_f1_1s.pl mod_Rep1_F1-S.bam all_rep_snpcall.txt &

perl new_extract_f1_2c.pl mod_Rep2_F1-C.bam all_rep_snpcall.txt &

perl new_extract_f1_2s.pl mod_Rep2_F1-S.bam all_rep_snpcall.txt &

5-2. extract parent read count

perl extract_apo_1c.pl mod_Rep1_APO-C.bam all_rep_snpcall.txt &

perl extract_apo_1s.pl mod_Rep1_APO-S.bam all_rep_snpcall.txt &

perl extract_apo_2c.pl mod_Rep2_APO-C.bam all_rep_snpcall.txt &

perl extract_apo_2s.pl mod_Rep2_APO-S.bam all_rep_snpcall.txt &

perl extract_ir64_1c.pl mod_Rep1_IR64-C.bam all_rep_snpcall.txt &

perl extract_ir64_1s.pl mod_Rep1_IR64-S.bam all_rep_snpcall.txt &

perl extract_ir64_2c.pl mod_Rep2_IR64-C.bam all_rep_snpcall.txt &

perl extract_ir64_2s.pl mod_Rep2_IR64-S.bam all_rep_snpcall.txt &

6. eXpress: gene expression level

Download:http://bio.math.berkeley.edu/eXpress/downloads/express-1.5.1/express-1.5.1-linux_x86_64.tgz

uncompress: tar -zxvf express-1.5.1-linux_x86_64.tgz

export to local workflow: export PATH=$PATH:/usrhome/LabLYLiu/r01621205//allelic_imbalance_project/expr4.0

6-1. Original gene expression level of APO, IR64, F1

#!/usr/bin/bash

for i in APO IR64 F1; do

express --output-dir express_Rep1_${i}_C modwithindel_ref.fasta mod_Rep1_${i}-C.bam &

express --output-dir express_Rep1_${i}_S modwithindel_ref.fasta mod_Rep1_${i}-S.bam &

done

for i in APO IR64 F1; do

express --output-dir express_Rep2_${i}_C modwithindel_ref.fasta mod_Rep2_${i}-C.bam &

express --output-dir express_Rep2_${i}_S modwithindel_ref.fasta mod_Rep2_${i}-S.bam &

done

6-2. read count of APO IR64 including SNP

samtools view -t modwithindel_ref.fasta.fai -Sb mod_Rep1_extract_APO_c.sam | express --output-dir rep1_express_e_apo_c modwithindel_ref.fasta - &

samtools view -t modwithindel_ref.fasta.fai -Sb mod_Rep2_extract_APO_c.sam | express --output-dir rep2_express_e_apo_c modwithindel_ref.fasta - &

samtools view -t modwithindel_ref.fasta.fai -Sb mod_Rep1_extract_APO_s.sam | express --output-dir rep1_express_e_apo_s modwithindel_ref.fasta - &

samtools view -t modwithindel_ref.fasta.fai -Sb mod_Rep2_extract_APO_s.sam | express --output-dir rep2_express_e_apo_s modwithindel_ref.fasta - &

samtools view -t modwithindel_ref.fasta.fai -Sb mod_Rep1_extract_IR64_c.sam | express --output-dir rep1_express_e_ir64_c modwithindel_ref.fasta - &

samtools view -t modwithindel_ref.fasta.fai -Sb mod_Rep2_extract_IR64_c.sam | express --output-dir rep2_express_e_ir64_c modwithindel_ref.fasta - &

samtools view -t modwithindel_ref.fasta.fai -Sb mod_Rep1_extract_IR64_s.sam | express --output-dir rep1_express_e_ir64_s modwithindel_ref.fasta - &

samtools view -t modwithindel_ref.fasta.fai -Sb mod_Rep1_extract_IR64_s.sam | express --output-dir rep2_express_e_ir64_s modwithindel_ref.fasta - &

6-3. extract F1 read count

samtools view -t modwithindel_ref.fasta.fai -Sb Rep1_from_apo_c.sam | express --output-dir rep1_express_f1_from_apo_c modwithindel_ref.fasta - &

samtools view -t modwithindel_ref.fasta.fai -Sb Rep1_from_ir64_c.sam | express --output-dir rep1_express_f1_from_ir64_c modwithindel_ref.fasta - &

samtools view -t modwithindel_ref.fasta.fai -Sb Rep1_from_apo_s.sam | express --output-dir rep1_express_f1_from_apo_s modwithindel_ref.fasta - &

samtools view -t modwithindel_ref.fasta.fai -Sb Rep1_from_ir64_s.sam | express --output-dir rep1_express_f1_from_ir64_s modwithindel_ref.fasta - &

samtools view -t modwithindel_ref.fasta.fai -Sb Rep2_from_apo_c.sam | express --output-dir rep2_express_f1_from_apo_c modwithindel_ref.fasta - &

samtools view -t modwithindel_ref.fasta.fai -Sb Rep2_from_ir64_c.sam | express --output-dir rep2_express_f1_from_ir64_c modwithindel_ref.fasta - &

samtools view -t modwithindel_ref.fasta.fai -Sb Rep2_from_apo_s.sam | express --output-dir rep2_express_f1_from_apo_s modwithindel_ref.fasta - &

samtools view -t modwithindel_ref.fasta.fai -Sb Rep2_from_ir64_s.sam | express --output-dir rep2_express_f1_from_ir64_s modwithindel_ref.fasta - &

6. Differential expression gene & Allelic imbalance test

R file : final.R
